# Anti-inflammatory effects of reactive oxygen species – a multi-valued logical model validated by formal concept analysis

**DOI:** 10.1186/s12918-014-0101-7

**Published:** 2014-09-24

**Authors:** Johannes Wollbold, Robert Jaster, Sarah Müller, Katja Rateitschak, Olaf Wolkenhauer

**Affiliations:** Department of Systems Biology and Bioinformatics, University of Rostock, Ulmenstr 69, D-18057 Rostock, Germany; Stellenbosch Institute for Advanced Study (STIAS), Wallenberg Research Centre, 10 Marais Street, Stellenbosch, 7600 South Africa; Department of Medicine II, Division of Gastroenterology and Endocrinology, University Medicine Rostock, E.-Heydemann-Str. 6, D-18057 Rostock, Germany

**Keywords:** Acute pancreatitis, Mitochondria, Reactive oxygen species, Calcium, Multi-valued logic, Formal concept analysis, Apoptosis, Necrosis

## Abstract

**Background:**

Recent findings suggest that in pancreatic acinar cells stimulated with bile acid, a pro-apoptotic effect of reactive oxygen species (ROS) dominates their effect on necrosis and spreading of inflammation. The first effect presumably occurs via cytochrome C release from the inner mitochondrial membrane. A pro-necrotic effect – similar to the one of Ca^2+^ – can be strong opening of mitochondrial pores leading to breakdown of the membrane potential, ATP depletion, sustained Ca^2+^ increase and premature activation of digestive enzymes. To explain published data and to understand ROS effects during the onset of acute pancreatitis, a model using multi-valued logic is constructed. Formal concept analysis (FCA) is used to validate the model against data as well as to analyze and visualize rules that capture the dynamics.

**Results:**

Simulations for two different levels of bile stimulation and for inhibition or addition of antioxidants reproduce the qualitative behaviour shown in the experiments. Based on reported differences of ROS production and of ROS induced pore opening, the model predicts a more uniform apoptosis/necrosis ratio for higher and lower bile stimulation in liver cells than in pancreatic acinar cells. FCA confirms that essential dynamical features of the data are captured by the model. For instance, high necrosis always occurs together with at least a medium level of apoptosis. At the same time, FCA helps to reveal subtle differences between data and simulations. The FCA visualization underlines the protective role of ROS against necrosis.

**Conclusions:**

The analysis of the model demonstrates how ROS and decreased antioxidant levels contribute to apoptosis. Studying the induction of necrosis via a sustained Ca^2+^ increase, we implemented the commonly accepted hypothesis of ATP depletion after strong bile stimulation. Using an alternative model, we demonstrate that this process is not necessary to generate the dynamics of the measured variables. Opening of plasma membrane channels could also lead to a prolonged increase of Ca^2+^ and to necrosis. Finally, the analysis of the model suggests a direct experimental testing for the model-based hypothesis of a self-enhancing cycle of cytochrome C release and ROS production by interruption of the mitochondrial electron transport chain.

**Electronic supplementary material:**

The online version of this article (doi:10.1186/s12918-014-0101-7) contains supplementary material, which is available to authorized users.

## Background

Acute pancreatitis is characterized by a rapid inflammatory process, which in the extreme case can lead to a systemic shock and to death. It is mainly caused by gallstones or alcohol. The severity of the disease depends on the proportion of apoptosis, autophagy and necrosis. The latter results in release of cellular constituents, damage of neighbouring cells and infiltration of pancreatic tissue with inflammatory cells [1]. In addition to the intracellular Ca^2+^ dynamics, reactive oxygen species (ROS) play a role in pancreatitis. Increases of ROS occur early in the disease, and preclinical antioxidant treatments significantly reduced pancreatic injury and inflammation. However, clinical tests of antioxidants produced conflicting results and were even stopped because of excess adverse events [2]. A pro-necrotic effect of ROS via opening of mitochondrial pores, loss of mitochondrial membrane potential and reduced ATP production is known. A new contribution of [2] was to show that specifically in pancreatic acinar cells this effect is dominated by an opposite pro-apoptotic effect, presumably via cytochrome C (Cyt c) release from the mitochondria [1].

With the present work, we investigate conditions for these opposite effects and test the hypothesis of Cyt c release. Therefore, we constructed a model to understand ROS regulation and ROS effects during the onset of acute pancreatitis. An increased knowledge of the pathophysiological role of ROS could contribute to the development of an antioxidant therapy which avoids negative side effects. Our original motivation was ageing research, more specifically the classical free radical theory of ageing (FRTA). It states that organisms age because cells accumulate damage by free radicals (or more generally ROS) and undergo senescence, apoptosis or necrosis [3]. During the last years, however, the FRTA was challenged [4], and more attention is given to physiological conditions where ROS have a protective role in signalling processes, for instance in response to molecular damage [5]. Our model describing one of these physiological ROS effects can account for a more differentiated understanding of real ROS effects on ageing.

**Figure 1:**
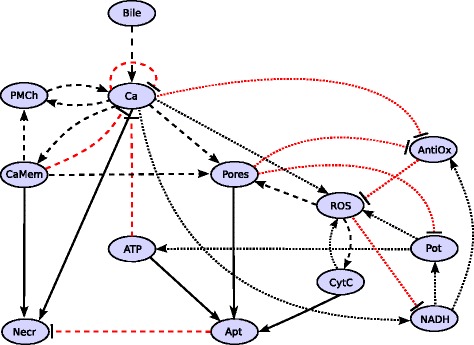
**Regulatory network underlying the logical functions and simulations.** Variables: *Bile* (TLC-S), *Ca* (Ca^2+^), *CaMem* (auxiliary variable representing a sustained Ca^2+^ increase), *PMCh* (plasma membrane channels), *NADH*, *Pot* (electric potential across the inner mitochondrial membrane), *ATP*, *ROS*, *AntiOx* (antioxidants neutralizing ROS), *CytC* (cytochrome C), *Pores* (mitochondrial permeability transition pores, MPTP, and mitochondrial outer membrane pores, MOMP), *Apt* (apoptosis, marked by caspase activation) and *Necr* (necrosis, marked by trypsinogen activation). Black arrows represent an activating influence, red lines with a bar inhibition. Dotted lines represent a short time scale of the interaction, dashed lines a larger one and continuous lines the largest time scale for the output variables.

The regulatory network of the model is shown in Figure 1. It is the result of an extensive literature search, the details of which are presented in Results and discussion. Here we give an overview on main processes.

The model was constructed to explain the data published in [2]. There, pancreatic acinar cells were stimulated with the bile acid taurolithocholic acid sulfate (TLC-S), reproducing a major cause of pancreatitis, the reflux of bile acid to the pancreas due to gallstones. Then, the changes of Ca^2+^, ROS, reduced nicotinamide adenine dinucleotide (NADH) and of the fraction of apoptotic/necrotic cells were measured. Since the hormone cholecystokinin (CCK) and the oligopeptide cerulein act analogously to TLC-S, we used respective experiments as confirmation of the observations in [2] or for proving the relevance of Ca^2+^ influx from the extracellular space [6].

It is known that TLC-S releases Ca^2+^ from the endoplasmatic reticulum (ER) and from acidic stores, probably the zymogen granula (ZG) containing the precursors of digestive enzymes, e.g. trypsinogen. Calcium loss from the ZG causes their disaggregation, which is necessary for the secretion of digestive enzymes to the duodenum, but in excess leads to their premature activation and ultimately to necrosis [7]. Ca^2+^ release can also trigger opening of mitochondrial pores, breakdown of the potential across the inner mitochondrial membrane and of adenosine triphosphate (ATP) production. Then Ca^2+^-ATP synthase (ATPase) is inhibited, which pumps Ca^2+^ back to the stores. The common opinion is that the sustained Ca^2+^ increase induced by this way is necessary for trypsin activation [1].

Directly and indirectly, an increase of cytosolic and subsequently mitochondrial Ca^2+^ concentration triggers the production of the ROS superoxide (O_2_^−^) at protein complexes of the electron transport chain (ETC) [8]. Different ROS molecules can easily be converted into each other, e.g. O_2_^−^ to hydrogen peroxide (H_2_O_2_). An increase of H_2_O_2_ solves the binding of Cyt c to the inner mitochondrial membrane, whereas hydroperoxyl (HO_2_) triggers pore opening at the outer mitochondrial membrane, comparable to Ca^2+^ effects. Then, Cyt c is released from the intermembrane space into the cytosol [9]. It activates the caspase signalling cascade leading to apoptosis and counteracting necrosis [10]. Note that we investigate a pathway of intrinsic, not extrinsic apoptosis mediated by death receptor activation. Furthermore, in [2] ROS did not enhance autophagy, which is why we do not take it into regard in our study.

The role of the auxiliary variable CaMem, of plasma membrane channels and of antioxidants is explained in Results and discussion.

In the literature, contradictory statements about interactions are found. For example, activating as well as inhibiting influences of Ca^2+^ on ROS are reported [8]. Furthermore, the ROS increase accompanied by a NADH decrease, which is measured in [2], is difficult to explain, since NADH fuels the electron transport, and ROS increase with membrane potential [11, p. 207, 210]. Even the central hypothesis of potential and ATP decrease during high stimulation is contested in [6, p. 13130].

This motivated a mathematical model to get a deeper understanding of the relevant processes and to guide the design of future experiments. In principle, systems of ordinary differential equations (ODE) can be very exact dynamical models. Detailed as well as simplified models with parameters based on experiments for various cell types exist for parts of our network, e.g. for Ca^2+^ handling [12], ATP and ROS production via the respiratory chain [11] or specific mitochondrial pores [13]. However, these different sources are difficult to unify and to integrate into a small model that is easy to understand. Moreover, only for a part of the relevant variables data were available for acinar cells stimulated with TLC-S. Therefore, we chose a qualitative representation of the essential dynamical features of this biological situation. A classical modelling method is a Boolean network introduced by S. Kauffman [14]. We use an extension to multi-valued logic propagated by R. Thomas [15,16].

During the process of model construction, we tested nearly 20 variants of logical equations in order to adapt the model to the data. Finally, we use Formal Concept Analysis (FCA) [17,18], a mathematical discipline in the domain of order theory. It is applied, e.g., in knowledge representation, information retrieval, ontology construction or data mining, but new in systems biology. We generate and visualize temporal rules for simulations, data and both, so as to validate the model against data and to gain further insights into the dynamics.

The outline of the paper is as follows. We will first explain the data available and then describe multi-valued logic, the asynchronous update scheme with three different time scales for fast, medium and slow processes, and the required methods from FCA (Methods). Results and discussion shows the network, the logical functions and compares simulation results with the data. It discusses the biological background and modelling decisions for calcium oscillations and sustained increase, ATP depletion and ROS production. The Conclusions summarize methods, results and open questions. Furthermore, the section gives an outlook on possible experimental tests of model predictions.

## Methods

### Data used for modelling

We used the following data to build our model:

- In [2], mouse (partly human) pancreatic acinar cells and isolated mitochondria were stimulated with 200/500 μM TLC-S. Ca^2+^, ROS, NAD(P)H were continuously measured for 10 and 20 minutes during stimulation, respectively (NADPH can be produced from NADH, and both are measured together). 30 minutes after the onset of stimulation, the proportion of apoptotic/necrotic cells was assessed by caspase activation and propidium iodide (PI) uptake, respectively.
- In [6], mouse acinar cells were stimulated with 10 pM and 10 nM CCK (acting analogously to TLC-S), and Ca^2+^ and trypsinogen activation was monitored.

If not stated explicitly, we hereafter refer to mouse data and to the experiments in [2].

### Formal characteristics of the multi-valued logical model

Our methodological framework is formal logic. Boolean networks describing biological systems were introduced in [14]. They are defined as a set of Boolean functions which determine the value of one variable (e.g. protein activated or not) at discrete time t + 1 according to the values of a subset of the network variables at t, connected by AND, OR and NOT. We use the extension of Boolean networks by multi-valued logic, which allows for a more fine-grained description of biological interactions.

**Figure 2:**
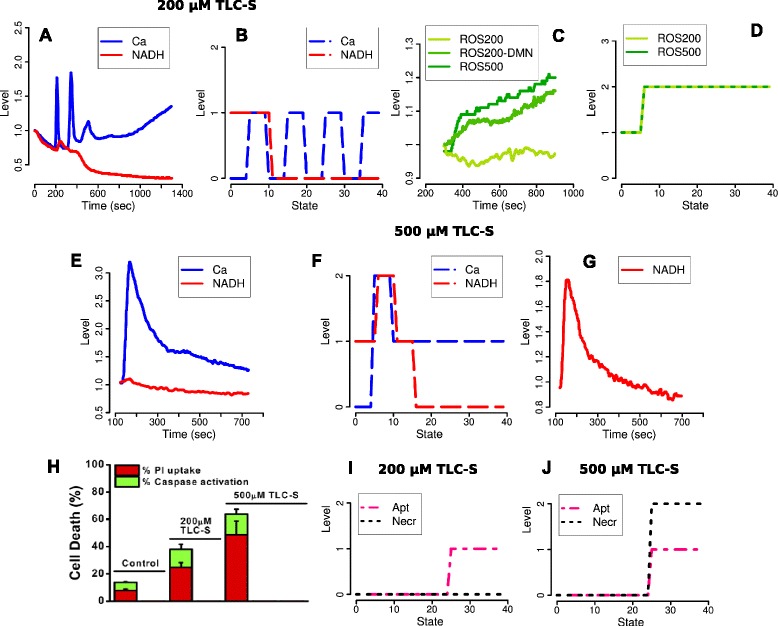
**Time profiles for the data and simulations during 200 μM and 500 μM TLC-S stimulation.** Our simulations reproduce main features of the measurements in [2]. **(** **A** **)** 25% of the murine pancreatic acinar cells show the mixed behaviour of a sustained increase of [Ca^2+^]_i_ after an oscillatory period, whereas our simulations **(** **B** **)** aim to reproduce the case of pure oscillations occurring for the majority of the cells (55%). **(** **E** **)**: The strong [Ca^2+^]_i_ peak after the onset of 500 μM stimulation followed by a sustained increase is mirrored by the simulations **(** **F** **)**. NAD(P)H decreases over the observation time. A first NAD(P)H peak during both stimuli **(** **A** **and** **E** **)** is weak for murine cells but strong for human acinar cells after the higher stimulation **(** **G** **)**. **(C** **)**: Intracellular ROS concentration increases for the higher but not the lower stimulation. Inhibition of the antioxidant NQO1 with 2,4-dimethoxy-2-methylnaphtalene (DMN) reveals that ROS production is also enhanced for 200 μM TLC-S. However, ROS are immediately scavenged by antioxidants, hence not released from the mitochondria. For both stimuli, the simulations **(** **D** **)** generate a similar ROS production defined as level 2, which is sufficient to induce CytC release and apoptosis. Compare the simulated time profiles **I** and **J** as well as **H**, the fraction of apoptotic cells measured by caspase activation, 30 min after the onset of the respective stimulation. Necrosis is marked by propidium iodide (PI) uptake. The *x*-axis of the simulations does not represent a fixed clock but the succession of logical states defined by possible changes in the values of the system variables. Pictures **A**, **C**, **E** and **G** are reproductions of the data shown in [2], **H** is adapted from ([2], Figure four C).

Data were discretized (Additional file 1) and possible values (levels) for not observed variables were chosen according to the criterion of potentially different biological causes or consequences. Hence, for the variable Bile we defined three levels for no, low (200 μM) and high (500 μM) stimulation with TLC-S, denoted in the format “2 Bile” for the logical functions of Table 1 and as “Bile.in.2”/“Bile.out.2” in the FCA rules described in Results. The Ca^2+^ measurement in Figure 2A was discretized to 0 – 1 – 0 – 1 – 0 – 1 – 1 (for 0, 100, 200, 300, 400, 600 and 1200 seconds after the onset of stimulation), since both the oscillation and the sustained increase at the end are judged as meaningful in the literature. Thus, for 11 variables (the meaning of which is indicated in Figure 1), we allow the three levels 0, 1 and 2: Bile, Ca, CaMem, NADH, Pot, ATP, AntiOx, Pores, CytC, Apt, Necr. For ROS, in addition the value 3 makes a difference: It can occur with inhibition of antioxidants (Table 1 (25)) and generates a stronger Cyt c release and subsequently apoptosis than 2 ROS (Table 1 (36), (38)). Only for PMCh, two levels were sufficiently expressive.

**Table 1:** **(Multi-valued) logical functions based on the network of Figure** 1

**ID**	**Input**		**Output**	**ID**	**Input**		**Output**
1	Bile_t_	=	Bile_t+1_	22	ROS_t_ + Pot_t_ + Ca_t_ + 2 !AntiOx_t_	=	2 ROS_t+1_
2	!Ca_t_ + Bile_t_	=	Ca_t+1_	23	ROS_t_ + CytC_t_ + 2 !AntiOx_t_	=	2 ROS_t+1_
3	PMCh_t_	=	Ca_t+1_	24	3 ROS_t_	=	2 ROS_t+1_
4	2 !ATP_t_	=	Ca_t+1_	25	2 ROS_t_ + Ca_t_ + CytC_t_ + !AntiOx_t_	=	3 ROS_t+1_
5	2 Ca_t_	=	Ca_t+1_	26	2 !Ca_t_	=	AntiOx_t+1_
6	!Ca_t_ + !CaMem_t_ + 2 Bile_t_	=	2 Ca_t+1_	27	2 !Pores_t_	=	AntiOx_t+1_
7	Ca_t_	=	CaMem_t+1_	28	2 AntiOx_t_	=	AntiOx_t+1_
8	2 Ca_t_	=	2 CaMem_t+1_	29	AntiOx_t_ + 2 NADH_t_ + !Ca_t_ + 2 !Pores_t_	=	2 AntiOx_t+1_
9	2 Ca_t_	=	PMCh_t+1_	30	Ca_t_	=	Pores_t+1_
10	2 !ROS_t_	=	NADH_t+1_	31	2 ROS_t_	=	Pores_t+1_
11	2 NADH_t_	=	NADH_t+1_	32	2 Pores_t_	=	Pores_t+1_
12	NADH_t_ + 2 Ca_t_	=	2 NADH_t+1_	33	Pores_t_ + Ca_t_ + CaMem_t_	=	2 Pores_t+1_
13	2 !Pores_t_	=	Pot_t+1_	34	2 ROS_t_	=	CytC_t+1_
14	2 Pot_t_	=	Pot_t+1_	35	2 CytC_t_	=	CytC_t+1_
15	Pot_t_ + 2 NADH_t_ + 2 !Pores_t_	=	2 Pot_t+1_	36	CytC_t_ + 3 ROS_t_	=	2 CytC_t+1_
16	!Pot_t_	=	ATP_t+1_	37	ATP_t_ + CytC_t_ + Pores_t_	=	Apt_t+1_
17	2 ATP_t_	=	ATP_t+1_	38	ATP_t_ + 2 CytC_t_ + Pores_t_	=	2 Apt_t+1_
18	ATP_t_ + Pot_t_	=	2 ATP_t+1_	39	Ca_t_ + CaMem_t_	=	Necr_t+1_
19	Pot_t_	=	ROS_t+1_	40	2 Ca_t_ + !(ATP_t_ + 2 CytC_t_ + Pores_t_)	=	2 Necr_t+1_
20	2 ROS_t_	=	ROS_t+1_	41	Ca_t_ + CaMem_t_ + !(ATP_t_ + 2 CytC_t_ + Pores_t_)	=	2 Necr_t+1_
21	ROS_t_ + 2 Pot_t_ + 2 !AntiOx_t_	=	2 ROS_t+1_				

The single equations determine the output at a simulation step t + 1, given the values of the variables at the input state (discrete time t). “!” means negation, “+” AND, OR is expressed by multiple equations for a specific level of the same output variable. Numbers denote the present value of a variable; 1 is not noted explicitly, 2 !A means “the value of A is below 2”. Hence, an equation !A_t_ + 2 B_t_ = C_t+1_ will be translated: If at a time point *t*, molecule/protein/process A is not activated/has a low concentration (hence A = 0) and at least a stimulus by B at level 2 is present (B_t_ ≥ 2), then at t + 1 the concentration of C is set to level 1. The function for the higher level of a variable is evaluated by priority. If no left-hand side of the respective equations is evaluated to TRUE, the default value of each variable is 0. This does not necessarily signify “zero” concentration, but a basic value which can be effective as in !Pot_t_, = ATP_t+1_. Temporal constraints like 2 Ca_t_ = Ca_t+1_ or Pot_t_ + .. = 2 Pot_t+1_ are not visualized in Figure 1.

Because the values observed in [2] are averaged over single cell measurements, we simulate the behaviour of single cells for 40 iterations, without considering cell communication. The logical functions of Table 1 generate transitions between states, given as vectors of variable values. The initial state is defined as the steady state before stimulation, with normal (1), inhibited (0) or enhanced antioxidant level (2) (Additional file 2). A synchronous update scheme would update all variables at every time step t. To reproduce the observed succession of events, it is necessary to discriminate between fast and slower processes. Fast processes include changes of membrane potential, ROS, antioxidants, NAD(P)H and ATP. The slower processes are Ca^2+^ and Cyt c release as well as opening of channels and pores. Fast processes are updated every single simulation step, while slower processes are updated every fifth step, after a stable value for the fast variables is reached. In addition, we introduce a third time scale for the modelling output, the initiation of apoptosis and necrosis as a function of the steady state of the remaining signalling variables. For the third time scale, 25 simulation steps were sufficient to reach a steady state.

The simulation steps do not denote fixed time intervals but logical steps indicating qualitative changes in the network state. Fast processes take maximally a few seconds. First, this group is comprised of the electro-chemical processes of ROS production and of a change in the mitochondrial membrane potential. For pancreatic beta cells, simulations in ([11], Figure seven) (with parameters derived from measured biophysical rates) show that the change of these two variables immediately follows an increase or decrease of glucose (hence NADH, via the citric acid cycle). ([19], Figure thirteen) simulates a comparably fast reaction of NADH and potential to a sudden increase in the intracellular calcium concentration [Ca^2+^]_i_, even though this reaction requires Ca^2+^ transport to the mitochondrial matrix (the space within the inner mitochondrial membrane). Another electro-chemical process is the activation of the antioxidant glutathione by NADH. It occurs via the supply of reducing equivalents (protons and electrons) [20, p. 208, 211]. Second, changes in the ATP concentration are slower but still in the range of a few seconds ([19], Figure thirteen). Third, loss of the antioxidant glutathione to the cytoplasm by concentration difference after pore opening [20, p. 210 f]. is rapid. Finally, we assume the inhibition of the glutathione related ROS removal system by Ca^2+^ [20, p. 208f., 210] as fast.

The larger time step of five units in the simulations is defined by the calcium dynamics: Steep Ca^2+^ increases occur 30 to 60 seconds after 500 μM stimulation, and the frequency of the Ca^2+^ oscillations after 200 μM stimulation is in the same range [2]. Medium stimulation of pancreatic mitochondria with 1.3 μM Ca^2+^ leads to pore opening and the onset of Cyt c diffusion to the cytosol after 1 min, reaching its maximum after 10 min ([10], Figure three A,C). The necessary condition of Cyt c diffusion, namely its release from the mitochondrial matrix via ROS, is set to the same time scale. Opening of plasma membrane channels prevents a complete Ca^2+^ decrease after the initial peak. This process occurs at the same slower time scale ([6], Figure two).

An upper limit for the meaning of the third time scale, the initiation of apoptosis and necrosis, is given by the respective measurements after 30 minutes in [2].

Generally, in one time step the value of a variable can only be increased or decreased by one, which is assured by equations like ATP_t_ + … = 2 ATP_t+1_. Rapid changes are allowed if there is evidence in the data. This is the case for the strong Ca^2+^ peak at the onset of the stimulation with 500 μM TLC-S. Furthermore, the value 2 of the output variables apoptosis and necrosis is determined by a single update.

Multi-valued logic is defined formally in [15] and has various implementations, e.g., CellNetAnalyzer [21]. We here use the notation of [21]. A multi-valued logical function is defined by Boolean functions for each level of the output variable. Take, for instance, the following statement (for the complete example see Table 1): “If the Ca^2+^ concentration is not high (not at discrete level 2), then antioxidants are produced (level 1)”. This is translated into the Boolean function:

$$ 2\kern0.5em !{\mathrm{Ca}}_{\mathrm{t}}={\mathrm{AntiOx}}_{\mathrm{t}+1} $$

The statement “If antioxidant production is medium (level 1) and the NADH concentration is high (level 2) and Ca^2+^ concentration is low (level 0) and pores are not opened completely (level 2), then antioxidant production is high”, is represented by:

$$ {\mathrm{AntiOx}}_{\mathrm{t}}+2\kern0.5em {\mathrm{NADH}}_{\mathrm{t}}+!{\mathrm{Ca}}_{\mathrm{t}}+2\kern0.5em !{\mathrm{Pores}}_{\mathrm{t}} = 2\kern0.5em {\mathrm{AntiOx}}_{\mathrm{t}+1} $$

Hence, + denotes AND, ! signifies NOT, Ca means Ca ≥ 1, 2 Ca is Ca ≥ 2, and NOT 2 Ca is expressed by 2 !Ca. In the case of competing functions (e.g. for Ca_t_ = 0), the Boolean function for the higher level of a variable is evaluated. OR is expressed by different equations for one level (thus, the left-hand sides of the equations together correspond to the disjunctive normal form of a logical clause):

$$ \begin{array}{l}!{\mathrm{Ca}}_{\mathrm{t}}+{\mathrm{Bile}}_{\mathrm{t}} = {\mathrm{Ca}}_{\mathrm{t}+1};\ 2\kern0.5em !{\mathrm{ATP}}_{\mathrm{t}} = {\mathrm{Ca}}_{\mathrm{t}+1}\\ {} \equiv \left(\mathrm{NOT}\kern0.5em {\mathrm{Ca}}_{\mathrm{t}}\kern0.5em \mathrm{AND}\kern0.5em {\mathrm{Bile}}_{\mathrm{t}}\right)\ \mathrm{OR}\kern0.5em \mathrm{NOT}\ 2\kern0.5em {\mathrm{ATP}}_{\mathrm{t}} = {\mathrm{Ca}}_{\mathrm{t}+1}\end{array} $$

We use knowledge assembled in the logical models [22] and [23] for the construction of our network. In [22], CellNetAnalyzer is employed for an analysis of the extrinsic and intrinsic apoptosis pathways. [23] unfolds an even broader “view of the interplays between NFkappaB pro-survival pathway, RIP1-dependent necrosis, and the apoptosis pathway in response to death receptor-mediated signals”. A main contribution of [23] is the reduction of a Boolean network in two steps resulting in a conceptual model with only three variables.

Because there is no logical model focusing on Ca^2+^, ROS, apoptosis and necrosis, particularly in the context of acinar cells with their specific ROS and mitochondrial pore regulation, for the present work, most interactions and logical functions had to be searched for in primary experimental literature or in biological reviews. Furthermore, we use knowledge from ODE models that describe electron transport, ROS production or Ca^2+^ dynamics [11,24].

CellNetAnalyzer [21] necessarily has limitations due to specific purposes and implementation choices. For instance, no auto-feedbacks are allowed, and we needed more flexible possibilities for an asynchronous update scheme. Therefore, we implemented the model in R [www.r-project.org], facilitating also further FCA analyses.

### Formal concept analysis

Simulations of the logical model, which was introduced above, generate transitions between states defined by the values of the variables at time t = 0, …, *n*. Referring to the example above, a simulation is realized by the evaluation of the Boolean functions according to the asynchronous update scheme, e.g. of 2 !Ca_t_ = AntiOx_t+1_. This generates a set of transitions, i.e. pairs of succeeding states:

$$ \left\{\left(\left({\mathrm{Ca}}_0,\dots, {\mathrm{AntiOx}}_0\right),\left({\mathrm{Ca}}_1,\dots, {\mathrm{AntiOx}}_1\right)\right),\dots ., \left(\left({{\mathrm{Ca}}_n}_{-1},\dots, {{\mathrm{AntiOx}}_n}_{-1}\right),\left({\mathrm{Ca}}_n,\dots, {\mathrm{AntiOx}}_n\right)\right)\right\} $$

Starting from this representation, Formal Concept Analysis (FCA) provides through *attribute exploration* an approach to discover logical rules that describe simulations and data. This helps to discern discrepancies between simulations and data. Furthermore, *concept hierarchies* are used to visualize dynamical features common to the simulations and data [18,25].

Starting from an initial condition, the model generates a sequence of states *s*_0_, *…, s*_*n*_ ∈ *S*, which is equivalent to a set of transitions {(*s*_0_*, s*_1_), (*s*_1_*, s*_2_), …, (*s*_*n*-1_*, s*_*n*_) | *s*_*i*_ ∈ *S* for all *i =* 0, *…, n*}. A state *s*_*i*_ is characterized by the value *f* ∈ *F =* {0,1,2,3} of each variable *e* ∈ *E* (entity, e.g. Ca, AntiOx), hence it is a vector (*f*_1_*, …, f*_|*E*|_) with *f*_*i*_ ∈ *F*. The set of the transitions for all investigated initial conditions is equivalent to a relation *R*_sim_ ⊆ *S* × *S* on the states. We are here interested in events that occur always, eventually or never. To this end, one determines the transitive closure *R*_sim_^trans^ of the relation *R*_sim_, which associates with a state any other successor state that may occur after an arbitrary number of time steps.

**Figure 3:**
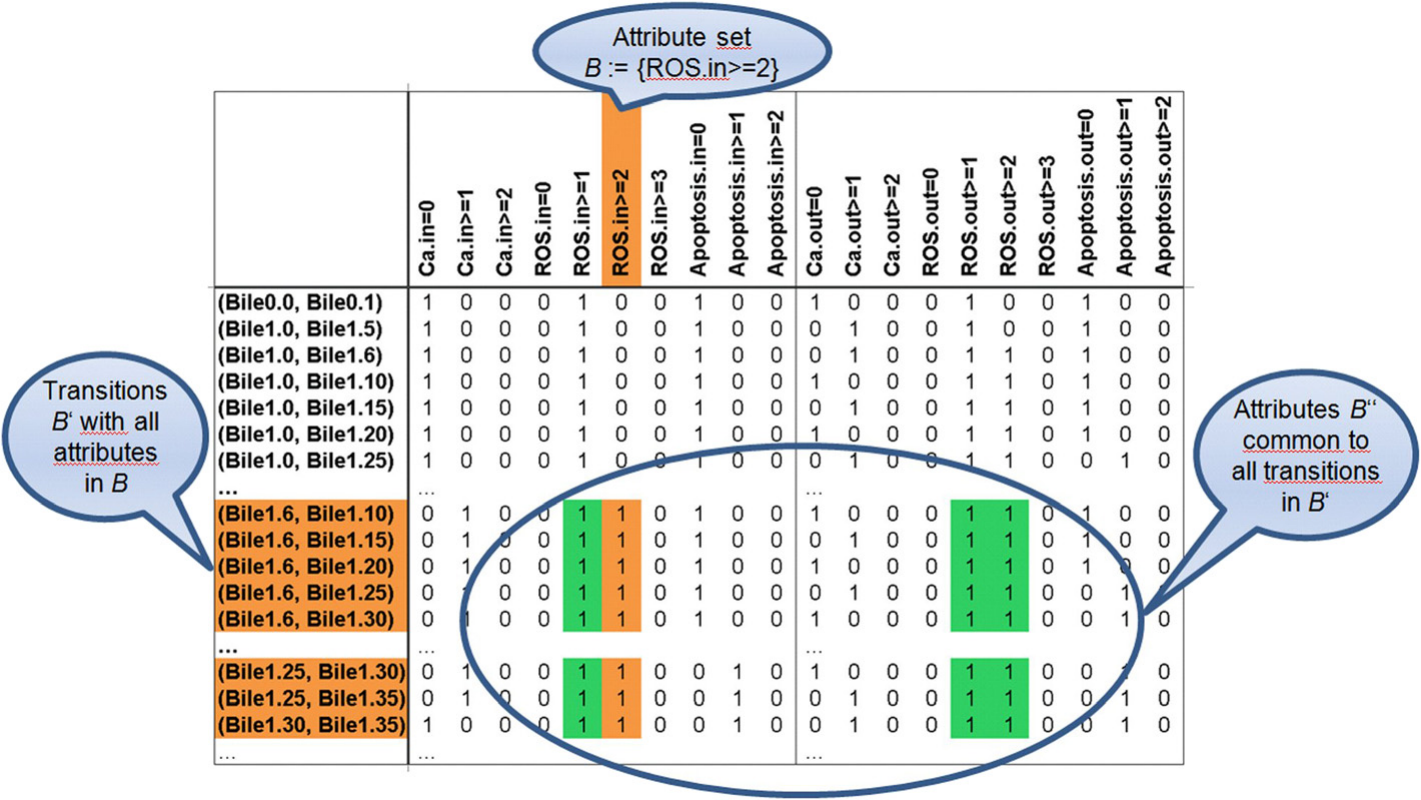
**Part of the transition context** ***K***_**sim**_ **for the simulations.** The rows are transitions between states following no (Bile0.*), low (Bile1.*) or high (Bile2.*) stimulation with TLC-S. Each (*input*) state is connected to any of the subsequent (*output*) states, yielding pairs like (Bile1.0, Bile1.1), (Bile1.0, Bile1.2), …, for the time points t = 0, 1, 2,… (transitions between two identical states are not shown). The attributes in the columns give the values of the variables in the input (*left*) and output (*right*) state. A formal concept is a maximal rectangle in a context, after a permutation of rows and columns. Hence, the orange and green cells represent the concept (*B*′*, B*′′). It is generated by the attribute set *B* := {ROS.in ≥ 2}: First, all transitions are assembled where ROS.in ≥ 2, then – in horizontal direction – all attributes common to these transitions, including remarkably ROS.out ≥ 2. This concept construction expresses that the ROS concentration does not decrease as soon as level 2 is reached. The concept also describes a transition set in the data.

In order to use FCA, we converted simulated time series into a *formal context*, specifically a *transition context K*_sim_ (Figure 3) introduced in [18]. This context is a data table defined as a triple (*G*_sim_*, M, I*_sim_), where the rows list the transitions *g* = (*s*_in_*, s*_out_) in *G*_sim_, which corresponds to the transitive closure *R*_sim_^trans^. Because FCA is based on binary data, the columns list the attributes *m* ∈ *M* in terms of the levels *f* a variable *e* can take, i.e. *M* ⊆ *E* × *F*. They indicate if a specific level of a variable is reached, or if its value is 0. For instance, in Figure 3, for ROS = 2 in the input state of the transition (Bile1.6, Bile1.10) we have the attributes ROS.in ≥ 1 and ROS.in ≥ 2. The elements of the table are then describing a relation *I*_sim_ ⊆ *G*_sim_ × *M.*

Analogously to the simulations, we computed for the data *R*_obs_, *G*_obs_ := *R*_obs_^trans^ and *K*_obs_ = (*G*_obs_*, M, I*_obs_) (Additional files 3 and 4) for several observed time series published in [2], after discretization into the same two to four levels *f* ∈ *F* (Additional file 1). The time steps for all experiments were chosen as 0, 100, 200, 300, 400, 600 and 1200 seconds. For all contexts, we selected the variable set *E* = {Bile, Ca, NADH, ROS, AntiOx, Apt, Necr} according to the availability of data. Transitions were generated for the conditions common to simulations and data: high, low and no TLC-S stimulation, without and with inhibition of antioxidants.

We combined *K*_sim_ and *K*_obs_ into a context *K*_com_ = (*G*_sim_ ∪ *G*_obs,_*M*, *I*_sim_ ∪ *I*_obs_) defined as the *subposition* of the two contexts. In fact, *K*_sim_ and *K*_obs_ are combined in one data table by union of the rows.

The field of FCA has developed mathematical tools [25] to understand relations *I* between various types of objects *G* and attributes *M*. The starting point are two derivation operators both denoted by ′. Given a formal context (*G, M, I*), the set of the attributes common to all objects in *A* ⊆ *G* is

$$ A\mathit{\hbox{'}}:=\left\{m\ \in\ M\mid \left(g,m\right)\ \in\ I\kern0.5em \mathrm{f}\mathrm{o}\mathrm{r}\ \mathrm{all}\ g\ \in\ A\right\}. $$

Dually, the set of the objects sharing all attributes in *B* ⊆ *M* is

$$ B\mathit{\hbox{'}}:=\left\{g\ \in\ G\mid \left(g,m\right)\ \in\ I\kern0.5em \mathrm{f}\mathrm{o}\mathrm{r}\ \mathrm{all}\ m\ \in\ B\right\}. $$

Then, a formal concept is a pair (*A, B*) with *A*′ *= B* and *B′ = A. A* is the *extent*, *B* the *intent* of the concept (*A, B*). (*A*′′*, A*′) and (*B*′*, B*′′) are always formal concepts. (*B*′*, B*′′) is constructed as follows (and (*A*′′*, A*′) analogously): For a given attribute set *B*, one considers the objects in *B*′ with at least these attributes, then the missing attributes common to these objects are added to *B*, yielding the *closure operator* ′′. This process can be visualized as drawing a maximal rectangle, starting from *B* (Figure 3). We will use the mode of expression “The concept (*B*′*, B*′′) is *generated* by the attribute (set) B”.

**Figure 4:**
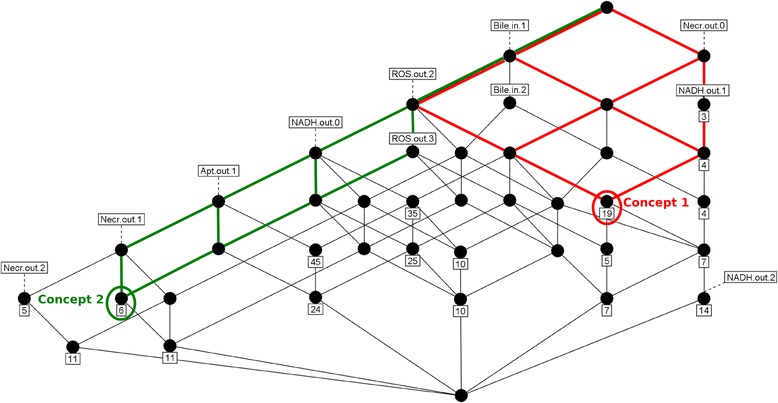
**Part of the concept hierarchy of the combined transition context K**_**com**_ **(cf. Figure** **3** **) for simulations and data.** Circles represent formal concepts, i.e. sets of transitions (pairs of input and output states) together with all their common attributes. The attributes of the input and output state can be read from the hierarchy by following the lines upwards. The concept order is defined by the subconcept – superconcept relationship: A superconcept is more general being determined by fewer attributes than a subconcept. At the same time, the superconcept’s object set (set of transitions) is enlarged. The number of objects that have exactly the respective attribute combination is indicated in the squared box below a concept. Hence, many general concepts in the upper part of the hierarchy have no own objects. As any formal concept, they embrace different more specific concepts below, characterized by supplementary attributes. A concept hierarchy is a complete and structured representation of a data set. The implications of the respective data set can be read from the hierarchy, and supplementary dynamical features can be identified. For examples and the meaning of the two highlighted concepts see Results and discussion.

Formal concepts can be ordered and visualized as a *concept hierarchy* (Figure 4). The order is defined by increasing generality. At the bottom of the hierarchy are the most specific concepts comprising few objects, on top of it are the most general concepts determined by only a few attributes: (*A, B*) ≤ (*C, D*), if *A* ⊆ *C* or equivalently *D* ⊆ *B.*

The structure of a concept hierarchy is also defined by implications *A → B* between attribute sets (for several examples of this connection see Results and discussion). They offer further insight into attribute dependencies, the more so as the information can be condensed into a minimal and complete (see below) rule set, the *stem base* of a given formal context. It can be computed interactively by the attribute exploration algorithm. Then an expert or a computer program accepts or rejects proposed implications. In the latter case, the expert has to provide a counterexample, i.e. a new object of the formal context. Because we validate an existing model and do not gather new dynamical rules, the automatic version of attribute exploration is more appropriate for our purpose, where every implication is accepted. We first computed the stem base of *K*_com_, the combined context for simulations and data. The resulting common, most reliable implications were an input to the exploration of *K*_sim_ and *K*_obs_, respectively (*background knowledge*). Their stem bases contain rules not implied by the background knowledge and mark dynamical differences between model and observations.

Completeness of the stem base means that queries regarding the validity of an arbitrary implication can be answered by logical inference from this minimal rule set. This compact representation of the logic of the three contexts by their stem bases enabled us to inspect them manually. This uncovered previously unnoticed dependencies of the temporal developments of several variables, for simulations, data and both. Highlighted dependencies between ROS, NADH, apoptosis and necrosis were analyzed deeper by a partial concept hierarchy for *K*_com_, which is also a complete representation of a data set. It is not as compact as the stem base, but enables fast visual inspection.

To generate the formal contexts from simulations and data, we programmed R scripts [www.r-project.org] available at www.sbi.uni-rostock.de/resources/software/. Concept hierarchies were visualized with Concept Explorer [http://sourceforge.net/projects/conexp/] and – allowing a better manual layout – Siena from the ToscanaJ suite [http://sourceforge.net/projects/toscanaj/]. Concept Explorer was also used to convert formal contexts from *.csv to *.cxt, readable by ConImp [www.mathematik.tu-darmstadt.de/~burmeister/] that computed the stem bases.

## Results and discussion

We first give an overview on the network structure and list the logical functions we arrived at as a result of the adaptation of the literature-based model to the data. Then, we will discuss literature findings regarding the main regulators Ca^2+^ and ROS and regarding their effects on apoptosis and necrosis, thus accounting for our modelling decisions. The fifth subsection gives the simulation results for 200 and 500 μM bile stimulation as well as for inhibition and addition of antioxidants. Furthermore we make predictions of the effect of a ROS burst observed in liver cells in contrast to pancreatic acinar cells. The following subsections contain the FCA model analyses. Finally, we will discuss an alternative model based on a minority position regarding a sustained Ca^2+^ increase and the initiation of necrosis.

### Biological interactions defining the network

Based on an extensive analysis of the literature related to pancreatic acinar cells (see Additional file 5), we assembled a regulatory network (Figure 1) for the variables Bile (TLC-S), Ca (Calcium Ca^2+^ ions), CaMem (an auxiliary variable representing a sustained Ca^2+^ increase by a simple “memory function”), PMCh (plasma membrane channels), NADH, Pot (electric potential across the inner mitochondrial membrane), ATP, ROS, AntiOx (antioxidants neutralizing ROS, e.g. glutathione (GSH) and NAD(P)H:quinone oxidoreductase 1 (NQO1)), CytC (Cytochrome c), Pores (mitochondrial permeability transition pores, MPTP, and mitochondrial outer membrane pores, MOMP), Apt (apoptosis, marked by caspase activation) and Necr (necrosis, marked by trypsinogen activation).

The network summarizes the regulation of apoptosis and necrosis in pancreatic acinar cells, stimulated with TLC-S, CCK or similar substances. The initial response to the stimulus is the release of Ca^2+^ from the ER and from ZG. Ca^2+^ is directly linked to one of the outputs of the network: A sustained increase of [Ca^2+^]_i_ leads to the activation of trypsin in the ZG and consequently to necrosis. Further primary effects of Ca^2+^ are the opening of (i.) Pores and (ii.) PMCh, (iii.) activation of NADH and (iv.) ROS, and (v.) decrease of AntiOx. Several closed loops offer insights to the progress of the system: The Ca-channel-loop consists of the Ca^2+^ induced activation of PMCh, which in turn keeps the Ca^2+^ level high. Three Ca-ATP-loops are regulated by the pores. Ca^2+^ induces pore opening directly and – less important – via ROS and AntiOx. Pore opening minimizes the mitochondrial membrane potential and consequently ATP supply. ATP decrease, in turn, inhibits Ca^2+^ storage to the ER and ZG. Hence, the Ca-ATP-loops represent a positive feedback on Ca^2+^ release. In the simulations performed, a negative feedback via NADH and ATP did not influence the Ca^2+^ dynamics. In addition, a negative auto-feedback of Ca^2+^ and a negative feedback via CaMem are introduced to model Ca^2+^ oscillations and the strong peak at the onset of high bile stimulation.

ROS production depends on Ca^2+^ and Pot. The membrane potential is also involved in two negative feedback loops starting from the ROS node. Positive feedbacks comprise NADH or Pores and AntiOx. Finally, inhibition of the electron transport via CytC detachment from the inner membrane is an important positive feedback on ROS production. CytC release via Pores leads to the activation of the caspase signalling chain and finally to apoptosis. Apoptosis and necrosis are alternative cell fates. While enhanced apoptosis reduces the fraction of necrotic cells, the reverse is not observed ([1], Figure four C).

The logical functions specifying the interactions discussed here are listed in the next section. A more detailed discussion of these interactions and reasons for their relevance in the context of the development of acute pancreatitis will be provided below.

### Multi-valued logical functions

The literature-based network was translated into a set of Boolean functions expressing multi-valued logic (Table 1). In Additional file 5, the degree of experimental confirmation is indicated: “experimentally confirmed” for most rules, “no experimental contradiction” for temporal constraints like (18) ATP_t_ + … = 2 ATP_t+1_, in contrast to (6) !Ca_t_ + !CaMem_t_ + 2 Bile = 2 Ca_t+1_ (compare the subsequent section), “auxililary rule” for the equations for CaMem. There are three “hypotheses based on experiments”, either since they are formulated as hypotheses in the literature (interaction Ca^2+^ → ROS) or since they represent our own interpretation of the data in [2] (2 !ROS_t_ = NADH_t+1_ and the prevalence of apoptosis over necrosis in equations (40) and (41)). Finally, equation (6) is a “phenomenological rule” generating the strong Ca^2+^ peak measured in [2] after 500 μM TLC-S stimulation. Additional file 5 lists also the alternative rules that specify one network interaction and that were tested during the process of model construction.

### Calcium oscillations, initial peak and sustained increase

Lower stimulations with TLC-S induce oscillatory Ca^2+^ signals spreading over the whole cell ([2,7], p. 1287, Figures two A and five). Respective models are based on different mechanisms [24, p. 9, Table one]. In our context, experimental evidence has suggested that shuffling of Ca^2+^ between the cytosol and the ER/ZG [7] and/or the extracellular space ([6], Figure two) plays a role ([26], p. 2206). Given that there is uncertainty about the relevant mechanisms, our model focuses on the Ca^2+^ dynamics and does not consider intra- or extracellular Ca^2+^ repositories as an explicit variable:

$$ (2)\ !{\mathrm{Ca}}_{\mathrm{t}} + {\mathrm{Bile}}_{\mathrm{t}} = {\mathrm{Ca}}_{\mathrm{t}+1} $$

The equation reads: If at a time point t = 1, at least a low bile stimulus is present (Bile_t_ ≥ 1) and [Ca^2+^]_i_ is low („!“ expressing negation, hence Ca = 0), then at t + 1 the calcium concentration is enhanced to level 1. Without TLC-S or after medium [Ca^2+^]_i_, intracellular calcium remains at or decreases to level 0.

The consequence of these oscillations is the initiation of apoptosis via Cyt c release from the inner mitochondrial membrane facilitated by ROS (Table 1, (34), (36), for the regulation of ROS see the next section). Partial opening of mitochondrial pores by ROS [9, p. 12768] as well as Ca^2+^ (Table 1, (30), (31)) enables the efflux of Cyt c to the cytosol, where it activates the caspase cascade [10, p. 437, 441] [27, p. 486], the decisive step towards apoptosis (Table 1, (37), (38)). The general Bax/Bak system is likely to be also involved in pore opening [10, p. 440]. Although it is important for extrinsic apoptosis mediated, for instance, by receptor binding of tumour necrosis factor α (TNF) [23], we do not consider it explicitly because there is no evidence that it is influenced by ROS or Ca^2+^ in the context investigated.

At the onset of 500 μM bile stimulation, a strong Ca^2+^ peak is measured [2]. We modelled this behaviour by another phenomenological rule:

$$ (6)\ !{\mathrm{Ca}}_{\mathrm{t}} + !{\mathrm{Ca}\mathrm{Mem}}_{\mathrm{t}} + 2\ {\mathrm{Bile}}_{\mathrm{t}} = 2\ {\mathrm{Ca}}_{\mathrm{t}+1} $$

The term !Ca_t_ + !CaMem_t_ requires a situation similar to the initial condition before bile stimulation, a steady state with low intracellular Ca^2+^ concentration. This initial steep increase of [Ca^2+^]_i_ is followed by a sustained increase, which initiates a vicious cycle: Mitochondrial pores open completely, the membrane potential breaks down, and ATP production is inhibited. Consequently, less Ca^2+^ is pumped into the ER (via ATPase), the ZG and through the plasma membrane ([7], p. 1286, 1290, Figure one). The latter three effects were subsumed by one inhibitory interaction ATP —| Ca^2+^. Hence, low ATP causes calcium increase and there is a self-sustaining cycle Ca^2+^ **→** Pores —| Pot **→** ATP —| Ca^2+^ (as well as two other feedback loops involving Ca^2+^ indicated above). Within our model, however, this cycle can only be initiated by a prolongation of a Ca^2+^ pulse. The missing link in the causal chain is provided by influx of extracellular Ca^2+^ through store-operated Ca^2+^ plasma membrane channels: Without extracellular Ca^2+^, the strong calcium increase after high CCK stimulation is shorter and trypsinogen, the precursor of the digestive enzyme trypsin, is not activated ([6], p. 13131, Figures two C, two G). In contrast, a sufficiently prolonged [Ca^2+^]_i_ elevation causes the release of trypsin already within the acinar cell, which thus digests itself and undergoes necrosis [7, p. 1286]. In parallel, ATP depletion shifts the balance from apoptosis to necrosis “via an inability of the apoptosome to activate the initiator caspase 9” [7, p. 1290]. We coded these effects into the following equations (Table 1):

- For the positive feedback of Ca and plasma membrane channels (PMCh):
  $$ \begin{array}{l}(9)\ 2\ {\mathrm{Ca}}_{\mathrm{t}} = {\mathrm{PMCh}}_{\mathrm{t}+1}\\ {}(3)\ {\mathrm{PMCh}}_{\mathrm{t}} = {\mathrm{Ca}}_{\mathrm{t}+1}\end{array} $$
- For the cycle Ca^2+^ **→** Pores —| Pot **→** ATP —| Ca^2+^:
  $$ \begin{array}{l}(33)\ {\mathrm{Pores}}_{\mathrm{t}} + {\mathrm{Ca}}_{\mathrm{t}} + {\mathrm{Ca}\mathrm{Mem}}_{\mathrm{t}} = 2\ {\mathrm{Pores}}_{\mathrm{t}+1}\\ {}(13)\ 2\ !{\mathrm{Pores}}_{\mathrm{t}} = {\mathrm{Pot}}_{\mathrm{t}+1}\\ {}(18)\ {\mathrm{ATP}}_{\mathrm{t}} + {\mathrm{Pot}}_{\mathrm{t}} = 2\ {\mathrm{ATP}}_{\mathrm{t}+1}\\ {}(4)\ 2\ !{\mathrm{ATP}}_{\mathrm{t}} = {\mathrm{Ca}}_{\mathrm{t}+1}\end{array} $$
- Throughout the simulations, a basic ATP level is assumed, even for low potential. It is necessary for the initiation of apoptosis:
  $$ \begin{array}{l}(16)\ !{\mathrm{Pot}}_{\mathrm{t}} = {\mathrm{ATP}}_{\mathrm{t}+1}\\ {}(37)\ {\mathrm{ATP}}_{\mathrm{t}} + {\mathrm{CytC}}_{\mathrm{t}} + {\mathrm{Pores}}_{\mathrm{t}} = {\mathrm{Apt}}_{\mathrm{t}+1}\end{array} $$

### Regulation of ROS production and NAD(P)H redox state

**Figure 5:**
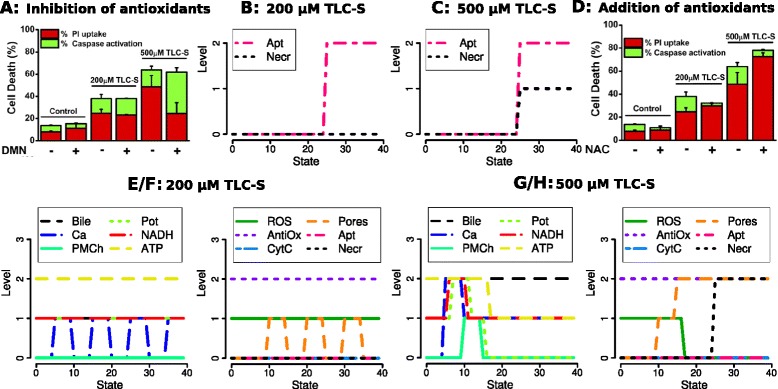
**Data (bar plots) and simulations for the inhibition and addition of antioxidants.** **A**: Fraction of apoptotic and necrotic cells with inhibition of the antioxidant NQO1 by DMN. **B** *:* Simulations during 200 μM bile (TLC-S) stimulation, **C** *:* Simulations during 500 μM TLC-S stimulation. For this condition, data and simulations exhibit increased apoptosis together with decreased necrosis (cf. Figure 2). The same enhancement of apoptosis is predicted already for 200 μM, whereas few changes are actually measured. **D** *–* **H** (addition of the antioxidant N-acetyl-l-cysteine (NAC)): In comparison to Figure 2, apoptosis decreases to level 0 for both stimulating conditions, but necrosis remains unchanged (0/2). In the simulations, NADH does not decrease. In contrast to Figure 2C, for 500 μM TLC-S stimulation ROS decrease to 0 after strong pore opening and potential breakdown. Figures *A* and *D* are reprinted from ([2], Figure four C and B), with permission from Elsevier.

In this section we justify the logical functions related to ROS and NAD(P)H. An important hypothesis analyzed by our model is: "Increases in ROS … promote apoptosis but not necrosis" [2, Abstract]. Accordingly, ([2], Figures one B, four A) (see our Figure 2C/H) shows an increase of ROS and enhanced apoptosis during 500 μM TLC-S stimulation (which only partly inhibits strong necrotic effects induced by Ca^2+^, cf. Figures 2 and 5). With 200 μM stimulation a slight decrease of cytosolic ROS is measured, nevertheless an almost equal fraction of cells as for the higher stimulation enters apoptosis. ([7], Figure one (c)) and ([2], Figure two B) reveal the influence of antioxidants: With the NQO1 inhibitor DMN, an increase of ROS is also measured after the lower stimulation, hence ROS production itself is enhanced. Obviously, ROS effects are partly independent from antioxidants, at least from NQO1 located in the cytosol.

ROS are mainly produced in the electron transport chain (ETC) of mitochondria and thus depend on the membrane potential ΔΨ: “As ΔΨ increases, it becomes more difficult for the ETC complexes to pump protons against that potential. Instead of completing their trip through the ETC, electrons in the ETC begin reacting directly with molecular oxygen, reducing it to form superoxide” [11, p. 210]. Ca^2+^ has antagonistic effects on ROS. On the one hand, it activates the citric acid cycle, which leads to the production of NADH, that in turn fuels the ETC [7, p. 1290] [11]. On the other hand, Ca^2+^ uptake by the mitochondrial matrix dissipates the membrane potential. This process tends to decrease ROS production and to enhance the oxidation rate of NADH [20, p. 210f.] [11, p. 207, p. 215 (32)]. According to ([11], Figure S four), NADH oxidation reaches a plateau for low potential, which according to our simulations only occurs during high bile stimulation at a late time point. Figure 2 shows that shortly after the onset of high (and possibly low) bile stimulation a small activating influence of Ca^2+^ on NADH and ROS prevails. The dynamics of these antagonistic effects could be represented exactly by an ODE model based, e.g., on [11]. We modelled the resulting general trend by the equations

$$ \begin{array}{l}(12)\ {\mathrm{NADH}}_{\mathrm{t}} + 2\ {\mathrm{Ca}}_{\mathrm{t}} = 2\ {\mathrm{NADH}}_{\mathrm{t}+1}\\ {}(15)\ {\mathrm{Pot}}_{\mathrm{t}} + 2\ {\mathrm{NADH}}_{\mathrm{t}} + 2\ !{\mathrm{Pores}}_{\mathrm{t}} = 2\ {\mathrm{Pot}}_{\mathrm{t}+1}\\ {}(21)\ {\mathrm{ROS}}_{\mathrm{t}} + 2\ {\mathrm{Pot}}_{\mathrm{t}} + 2\ !{\mathrm{AntiOx}}_{\mathrm{t}} = 2\ {\mathrm{ROS}}_{\mathrm{t}+1}.\end{array} $$

So far, we have not yet explained the observed sustained ROS increases. This could neither be achieved by modelling loss of the antioxidant glutathione via mitochondrial pores, nor by inhibition of the glutathione related ROS removal system via Ca^2+^ [20, p. 210f.] (Table 1 (29)). Furthermore, ROS can be generated directly by NADPH oxidase, but this enzyme is not present in pancreatic acinar cells [1, p. 2124]. However, we introduced a direct influence of Ca^2+^ on ROS, under the condition of normal electron transport (Pot = 1) [8, p. C1083]:

$$ (22)\ {\mathrm{ROS}}_{\mathrm{t}} + {\mathrm{Pot}}_{\mathrm{t}} + {\mathrm{Ca}}_{\mathrm{t}} + 2\ !{\mathrm{AntiOx}}_{\mathrm{t}} = 2\ {\mathrm{ROS}}_{\mathrm{t}+1} $$

The influence of Ca on ROS was confirmed by the rule resulting from the attribute exploration of simulations and data (see below and Additional file 4, Kcom.pro (38)):

$$ \mathrm{C}\mathrm{a}.\mathrm{in}.2\ \to\ \mathrm{C}\mathrm{a}.\mathrm{out}.1,\ \mathrm{R}\mathrm{O}\mathrm{S}.\mathrm{out}.2. $$

**Figure 6:**
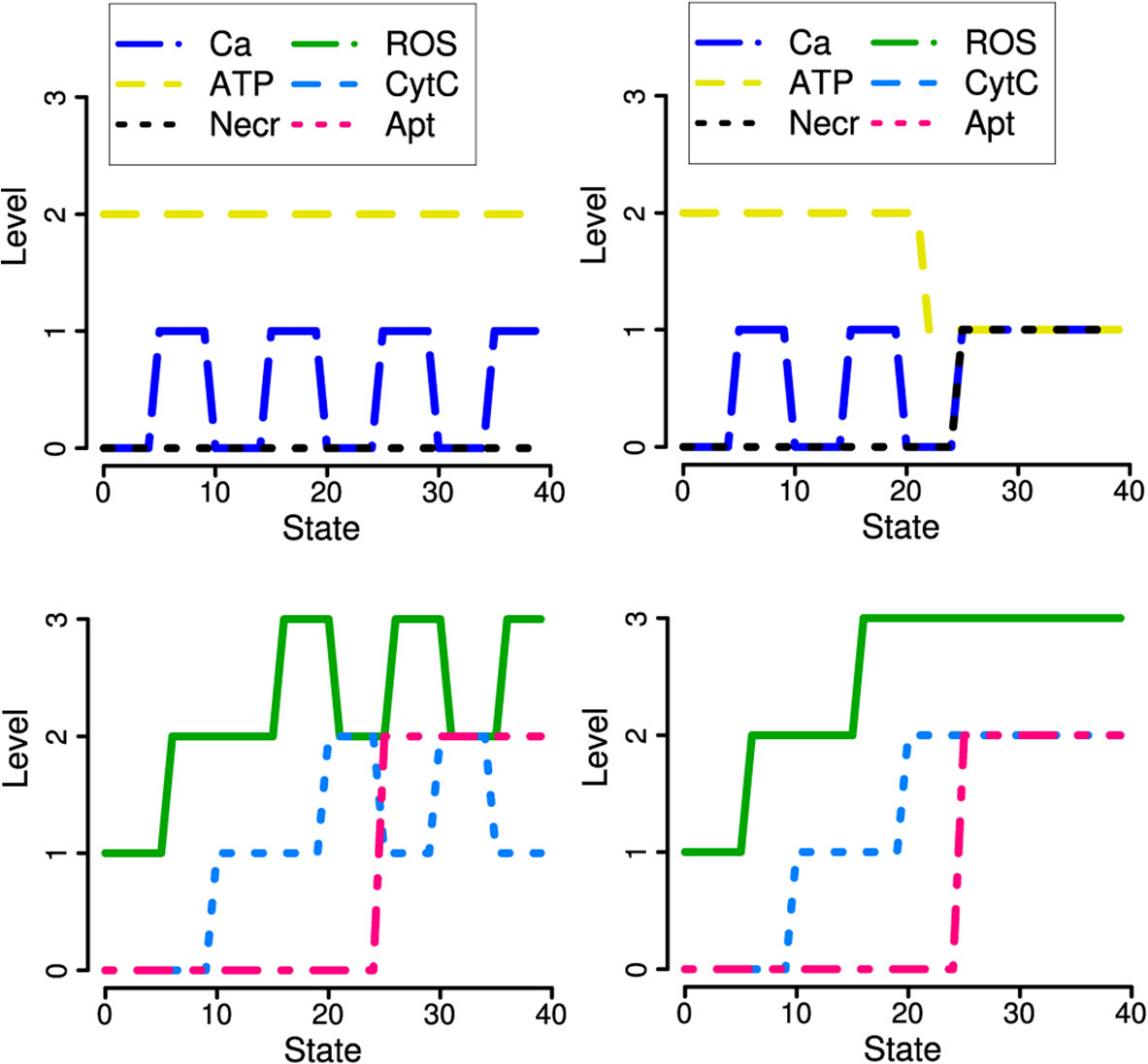
**Simulations for 200 μM TLC-S stimulation of acinar (** ***left*** **) and liver cells (** ***right*** **).** Inhibition of antioxidants enables ROS increase up to the maximal level 3. Liver cells are reported to be more sensitive to ROS induced mitochondrial depolarization, pore opening, ATP depletion and subsequent necrosis [1]. Thus, ROS have a role similar to the role of Ca^2+^ in acinar cells stimulated with 500 μM TLC-S (cf. Figure 2).

To capture the experimentally observed dynamics, it was necessary to introduce still another effect. This led us to a rule about an effect of the observed dissociation of Cyt c from the membrane. This process inhibits the electron transport, increases the electron concentration in the mitochondrial redox centers and ultimately leads to ROS production similar, e.g., to the complex I inhibitor rotenone [28]. Within the model framework, already for 200 μM bile stimulation this effect is necessary to induce the measured fraction of apoptotic cells. During the Ca^2+^ peak of the first oscillation, ROS increase to level 2. Ca^2+^ oscillates on the slower time scale with changes in the range of one minute. This relatively short increase is supposed to be sufficient to release Cyt c from the inner mitochondrial membrane, in accordance with ([10], Figure three A). Then, electron transport is inhibited, and ROS production and Cyt c release maintain each other in a positive feedback loop, leading finally to apoptosis (cf. Figure 6 and Additional file 2). This hypothesis should be tested experimentally. It becomes more plausible, if one assumes a slower mutual enhancement of ROS production and Cyt c release during several oscillatory cycles of Ca^2+^. However, this would complicate the model further by the introduction of supplementary levels and auxiliary variables for time delays.

Summarizing the regulation of ROS (besides the obvious influence of antioxidants), our model represents a primary effect of Ca^2+^ on ROS according to equations (22) (see above) and – via NADH and membrane potential – (21) (Table 1). Enhanced ROS production (level 2) maintains itself via ROS → Cyt C release → blocking of electron transport → ROS:

$$ \begin{array}{l}(34)\ 2\ {\mathrm{ROS}}_{\mathrm{t}} = {\mathrm{CytC}}_{\mathrm{t}+1}\\ {}(23)\ {\mathrm{ROS}}_{\mathrm{t}} + {\mathrm{CytC}}_{\mathrm{t}} + 2\ !{\mathrm{AntiOx}}_{\mathrm{t}} = 2\ {\mathrm{ROS}}_{\mathrm{t}+1}\\ {}(25)\ 2\ {\mathrm{ROS}}_{\mathrm{t}} + {\mathrm{Ca}}_{\mathrm{t}} + {\mathrm{CytC}}_{\mathrm{t}} + !{\mathrm{AntiOx}}_{\mathrm{t}} = 3\ {\mathrm{ROS}}_{\mathrm{t}+1}\end{array} $$

Finally, we will discuss the regulation of NADH, which is closely related to ROS. In the argument above for equation (12), which generates an initial peak of NADH, we already mentioned the activating influence of Ca^2+^ via the TCA cycle [7,11]. This activation of NADH can be supported by inhibition of NADH oxidation as a consequence of the ETC disruption by Cyt c release [28, p. 548]. However, the observed long term behaviour for low and high TLC-S stimulation is NAD(P)H decrease (Figure 2). For the higher stimulation, this could be explained by loss of glutathione to the cytosol via pore opening, which is counteracted by delivering of reducing equivalents from NADH via NADPH to glutathione [20, p. 208, 210f]. For both stimuli, we use this important role of NAD(P)H within the antioxidant system as a more general argument: With increasing ROS more reducing equivalents for antioxidants are needed, and indeed supplied by the NADH pool. Hence we defined the following equation, in accordance with the strong negative correlation between ROS and NADH measured in [2]:

$$ (10)\ 2\ !{\mathrm{ROS}}_{\mathrm{t}} = {\mathrm{NADH}}_{\mathrm{t}+1}. $$

From this equation follows that, if ROS are present at a certain level (2 or more in our representation), then the level of NAD(P)H is decreased to 0 (unless equations (11) and (12) apply temporarily).

### Simulation results

We performed simulations starting from normal physiological conditions without, with low and high bile stimulation, furthermore with inhibition or addition of antioxidants. Finally, two logical functions were changed in order to predict the behaviour of liver cells, where a higher ROS sensitivity of mitochondrial pores and a ROS burst after pore opening are reported [1,10].

In the following, we will compare simulation results for main processes with the data. For the complete simulations see Additional file 2, for the discretized data from [2] see Additional file 1.

#### Main condition: Stimulation with bile acid

The following time profiles are reproduced by our simulations (Figure 2):

- [Ca^2+^]_i_ oscillations occur during 200 μM bile stimulation for 55% of the cells (for another 20%, there is a sustained [Ca^2+^]_i_ increase, and 25% show the mixed behaviour of Figure 2A).
- All cells exhibit a strong [Ca^2+^]_i_ peak after the onset of 500 μM stimulation, followed by a sustained increase (Figure 2E/F).
- There is a pronounced NAD(P)H peak after the onset of 500 μM stimulation for human cells (Figure 2F/G), then NAD(P)H decreases for both cell types and stimuli (Figure 2A/B/E-G).
- For both stimuli, a medium fraction of all cells undergoes apoptosis (compared to the inhibition of antioxidants and 500 μM TLC-S, see below). There is strong necrosis for 500 μM TLC-S. In accordance with [2], we conclude from the observations reproduced in Figure 2H, that there is almost no necrosis for the modelled case of pure Ca^2+^ oscillations with 200 μM TLC-S stimulation.
- ROS production but not release from the mitochondria increases for both stimuli.

#### Inhibition and addition of antioxidants

Another notable result is that apoptosis increases and necrosis decreases for 500 μM bile stimulation, if antioxidants are deactivated. This observation is reproduced by the respective simulation (Figure 5). The same enhancement of apoptosis is predicted already for 200 μM due to increased ROS release to the cytosol, whereas few changes are actually measured.

For the addition of the antioxidant NAC, only data for apoptosis and necrosis were published in [2]. Our simulations reproduce the decrease of apoptosis for both stimulating conditions. An increase of necrosis during 500 μM bile is not reflected by the simulation, since already without NAC the maximal level of Necr = 2 is reached (Figure 2).

Further predicted differences to the basic conditions are:

- NADH does not decrease (cf. Figure 2), since the inhibitory influence of ROS is missing.
- Pore opening oscillates dependent on Ca^2+^, since the activation by 2 ROS is missing (Table 1, (30) and (31)).
- Antioxidants inhibit the initial increase of ROS (Table 1, (21), (22)), subsequent CytC release (34) and positive feedback on ROS production (23). Moreover, the ROS level decreases to 0 after strong pore opening and potential breakdown (19) induced by 500 μM TLC-S.

### Prediction: ROS burst in liver cells

Pancreatic acinar cells have specific properties compared to other cells like liver cells. In order to simulate their behaviour, we made the following changes of the logical functions:

- Excessive opening of mitochondrial pores (MPTP) leads to swelling of the mitochondrial matrix, rupture of the outer mitochondrial membrane (OMM) and a ROS burst, possibly caused by loss of antioxidants [10, p. 440]. This is modelled by a new rule:
  $$ 2\ {\mathrm{ROS}}_{\mathrm{t}} + 2\ {\mathrm{Pores}}_{\mathrm{t}} + 2\ !{\mathrm{AntiOx}}_{\mathrm{t}} = 3\ {\mathrm{ROS}}_{\mathrm{t}+1} $$
- The ROS burst is supposed to cause the strong Cyt c release observed in liver cells under the same conditions [10, p. 431, 440]. This follows according to the unchanged rule CytC_t_ + 3 ROS_t_ = 2 CytC_t+1_.
- Higher sensitivity of mitochondrial depolarization to ROS is introduced to the model by Pores_t_ + 3 ROS_t_ = 2 Pores_t+1_ [2, p. 1879].
- The Ca^2+^ – necrosis interaction by trypsin activation is specific for acinar cells. It is mainly influenced by ATP depletion, and for liver cells is replaced by a direct inhibitory influence of ATP on necrosis [29]:
  $$ \begin{array}{l}2\ !{\mathrm{ATP}}_{\mathrm{t}}={\mathrm{Necr}}_{\mathrm{t}+1}\\ {}2\ !{\mathrm{ATP}}_{\mathrm{t}} + !\left({\mathrm{ATP}}_{\mathrm{t}} + 2\ {\mathrm{CytC}}_{\mathrm{t}} + {\mathrm{Pores}}_{\mathrm{t}}\right)=2\ {\mathrm{Necr}}_{\mathrm{t}+1}\end{array} $$
  The term in brackets is the condition for apoptosis. Hence, we assume a prevalence of apoptosis over necrosis, as it is shown for acinar cells in [2] (compare Figure 5A).
- The bile – Ca^2+^ interaction remained unchanged but is interpreted as an analogous stimulus, e.g. copper exposure [30] or ischemia-reperfusion injury after heart failure [29].

**Figure 7:**
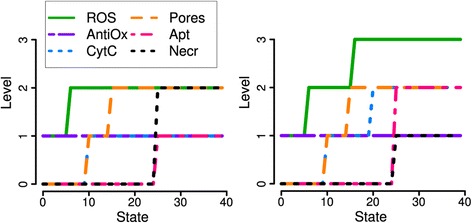
**Simulations for 500 μM TLC-S stimulation of acinar (** ***left*** **) and liver cells (** ***right*** **).** A ROS burst following strong opening of mitochondrial pores causes enhanced apoptosis and decreased necrosis (cf. Figure 2).

This resulted in the following modifications of cell fate:

- For high stimulation, the ROS burst causes enhanced apoptosis and decreased necrosis (Figure 7) for liver cells, which is in agreement with [10, p. 440]. However, the Ca^2+^ and ROS measurements of [2] and accordingly our simulations do not support the stronger hypothesis regarding pancreatic mitochondria, based on Ca^2+^, potential, ROS and Cyt c measurements: “mPTP opening […] blocks ROS production, thus inhibiting cytochrome c release” [10, p. 440] and subsequently apoptosis.
- With inhibition of antioxidants, pore opening, ATP depletion and necrosis occur already for a lower stimulation, via ROS (Figure 6). Due to Ca^2+^ oscillations, no steady state is reached for ROS and CytC in the pancreas simulation. Therefore, depending on the time step for apoptosis, its value is predicted as one (corresponding to [2]) or two. This can be interpreted as an intermediate value, hence slightly reduced apoptosis in pancreatic cells.
- Since with addition of antioxidants ROS levels do not increase above 1, no difference between liver and pancreatic cells is predicted.

In summary, there is a more uniform apoptosis/necrosis ratio for lower and higher stimulation, without and with inhibition of antioxidants. This can be tested by parallel experiments for both cell types with stimuli inducing Ca^2+^ oscillations and a sustained increase, respectively.

### Comparison of simulations and data by attribute exploration

As described in the Methods section, we translated the modelled and observed dynamics into three transition contexts *K*_obs_, *K*_sim_ and *K*_com_, which are tabular structures for the data (Additional files 3 and 4), the simulations (Figure 3) and their combination. The rule set of the combined context *K*_com_, computed by the attribute exploration algorithm, is most trustable because these rules are obviously valid in both contexts *K*_sim_ and *K*_obs_. Other rules that only hold in one of the two first contexts highlight differences between the literature-based model and the data. For the comparison of simulations and data, we selected the variables Bile, Ca, NADH, ROS, Apt and Necr, for which data are available without, with low and high bile stimulation. AntiOx values are supposed to remain stable as in the simulations. For inhibition of antioxidants, apoptosis, necrosis and ROS were measured in [2]. Since Bile is the input variable and Ca/NADH are not influenced by inhibition of antioxidants according to the simulations, we also included the respective measurements in *K*_obs_.

Rules relate to any input state occurring during an experiment or simulation, and/or an arbitrary subsequent (output) state. They describe what happens in parallel, always before or always after an event, or eventually depending on supplementary conditions.

For the combined simulation and data context *K*_com_, 90 rules were generated (see Additional file 4). These include several interesting findings that are not obvious from the visual inspection of the simulated and observed time series under different conditions (Figure 2 and Figure 5). For illustration purposes, we describe three rules. ‘,’ means AND.

Necr.out.2 → Apt.out.1: In an output state, strong necrosis always occurs together with at least medium apoptosis. A second rule, Apt.out.1 → NADH.out.0, ROS.out.2, highlights preconditions for apoptosis: If medium apoptosis is initiated, NADH is low and the ROS concentration is at level 2. Moreover, ROS ≥ 2 for states immediately before Apt = 1 (compare to Figure 2, for the basic experimental conditions). Thus, the logical functions implementing the causal relationship 2 ROS – CytC – Apt (Table 1, (34) – (38)) are compatible with the data. Finally, two rules highlight details of the mutual dependency between NADH and ROS discussed above. According to NADH.out.0 **→** ROS.out.2 and NADH.out.2 **→** ROS.out.2, Bile.in.2, concentrations of ROS are elevated during the NADH peak after 500 μM stimulation (Bile.in.2) and when NADH production is decreased, not necessarily during the intermediate condition.

In addition to the 90 rules of *K*_com_, attribute exploration generated 10 rules that hold for the simulations but not the data. These rules could provide valuable hints for further improvements of the model or they could provide the basis for new hypotheses. Thus, the rule Necr.out.1 **→** Bile.in.2 states that high stimulation with TLC-S is a necessary precondition for necrosis to be initiated. The occurrence in this rule set referring to the simulations is due to the fact that we excluded from our simulations the intermediate case of Ca^2+^ oscillations and subsequent sustained increase during 200 μM TLC-S (cf. Figure 2A). In the data, the respective transitions with attributes Necr.out.1 and Bile.in.1 are counterexamples to the rule, hence it is not included in the rule base common to data and simulations. For the simulated sustained oscillations, however, (corresponding to 55% of the cells, see the legend of Figure 2) there is no necrosis when Bile = 1. This could provide an interesting hypothesis for further experimental validation. Likewise, for Ca.in.0, ROS.in.2 **→** NADH.out.0, Necr.out.0 there is a counterexample in the data. The premise holds for several states during medium stimulation and Ca^2+^ oscillations. The rule expresses that in all future states the NADH concentration remains low, and necrosis is not initiated. In the data, the first conclusion also holds. Necrosis, however, occurs in the intermediate case excluded from our considerations. Third, consider ROS.in.2, NADH.out.1 **→** Ca.in.1: In states characterized by ROS ≥ 2 with subsequent NADH level of 1, Ca is at least at level 1. There is no qualitative difference of simulations and data, but for 200 μM TLC-S, ROS increase earlier in the data, i.e. (before and) during the first oscillatory Ca^2+^ decrease (Ca.in.0), whereas NADH remains at level 1 until the second Ca^2+^ increase (Figure 2 and Additional file 3).

30 rules are valid for the data, not for the simulations. Complementary to the previous rule set, these rules show where the simulations do not follow strictly the specific data set and highlight explicit or implicit model hypotheses. An example is Ca.in.1, ROS.in.3 **→** NADH.out.0. The rule points at a slight difference in the succession of events. In the simulations, for a single transition NADH.out.1 is true after Ca.in.1 and ROS.in.3 (cf. ROS.in.2, NADH.out.1 **→** Ca.in.1 valid for the simulations). Given the time discretization in intervals of 100 seconds, the following two rules show an equivalence between Ca and NADH: NADH.out.2 → Ca.in.0, Ca.out.2 and Ca.out.2 → NADH.out.2. In the simulations, however, NADH follows Ca after one time step (cf. Figure 2). This asynchronicity results from a more detailed view on the process, since we assume an activating influence of Ca on NADH (Table 1, (12)). Similarly, the rule NADH.in.0, Ca.out.0 **→** Apt.out.1 points at a subtle difference in the dynamics: If after NADH decrease a Ca minimum is reached, apoptosis is initiated (cf. Figure 2). In the simulations, NADH decrease occurs earlier, hence a supplementary oscillation is possible until apoptosis is initiated.

### A concept hierarchy illustrates dynamical features of the data and the model

Finally, we visualized the first rule set described in the previous section and made a more detailed dynamical analysis for selected cases. Our purpose was to generate a structured and complete representation of the dynamics of the output variables apoptosis and necrosis, together with their regulator ROS and its relation to NADH, which was highlighted by the rule set. We computed the concept hierarchy of the combined transition context for simulations and data (see Methods). All three implications listed in the previous section and which are common to simulations and data can be read from the subgraph of the concept hierarchy (Figure 4), via the order of attributes and attribute combinations. For instance, the concept generated (see Methods and Figure 3) and labelled by NADH.out.0 is below the concept generated by ROS.out.2. This means that all transitions with attribute NADH.out.0 have also the attribute ROS.out.2. Therefore, the implication NADH.out.0 **→** ROS.out.2 is valid. Furthermore, one can read the rule NADH.out.2 **→** ROS.out.2 from the hierarchy. The two implications express that ROS react faster than NADH: If the start level 1 of NADH has changed, ROS concentration has increased to at least 2. The inverse implication, however, is not valid.

The implication NADH.out.1 **→** ROS.out.2 does not apply. Yet there are several output states where NADH remains unchanged (level 1) but ROS have increased. These states are the objects of the most superordinate and general formal concept that has the attributes ROS.out.2 and NADH.out.1 (Concept 1 in Figure 4). In addition, this concept has all other attributes above, i.e. Bile.in.1 and Necr.out.0. The hierarchical status of the latter attribute above NADH.out.1 indicates the validity of the implication NADH.out.1 **→** Necr.out.0. Thus, NADH ≥ 1 only holds before the initiation of necrosis.

Several concepts with the attribute ROS.out.2 show two further rules mentioned in the previous section, Necr.out.2 **→** Apt.out.1 and Apt.out.1 → ROS.out.2, in addition the rule inferred from these Necr.out.2 **→** ROS.out.2. This rule signifies that Necr ≥ 2 is a subcase of ROS ≥ 2. In spite of the induction of apoptosis, this ROS level is not sufficient to stop strong necrosis.

In contrast, a maximal ROS level keeps necrosis at a medium level. Consider Concept 2 in Figure 4: It is generated by Necr.out.1 and ROS.out.3 and is a subconcept of the one generated by ROS.out.2. The concept above, labelled Necr.out.1, has no own objects (see legend to Figure 4), just as its subconcept right of Concept 2. Therefore, all transitions where Necr ≥ 1 but not ≥ 2 are assembled in Concept 2. The hierarchy reveals that the transitions of this concept not only have the attribute ROS.out.2 but necessarily ROS.out.3. Hence, for simulations and data reduced necrosis is connected with the highest ROS level (induced by inhibition of antioxidants). This underlines the protective role of ROS.

### ATP depletion

Because of the discussion concerning ATP depletion in the literature, we investigated a variation of the main model but without ATP depletion. In our model, we explain the sustained [Ca^2+^]_i_ increase during stimulation with 500 μM TLC-S by depolarization of mitochondria. This effect is contested by [6, p. 13130] for acinar cells stimulated by cerulein, whereas ([7], Figure one for TLC-S and Figure four for palmitoleic acid) and [31] (for ATP measurements in vivo after 24 hours) confirm the interpretation of [2] and [1]. Our own ATP measurements during 200 and 500 μM TLC-S stimulation did not prove a pronounced decrease of ATP (data not shown).

**Figure 8:**
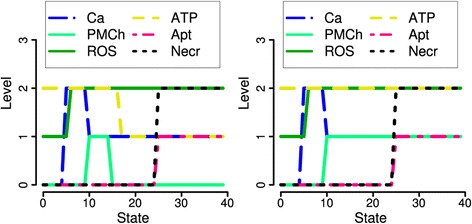
**Modelling high bile stimulation with (left) and without (right) the assumption of ATP depletion.** In our standard model (*left*), an ATP decrease to 1 prolongs medium [Ca^2+^]_i_ (level 1) sufficiently long to induce necrosis. In the alternative model (*right*), this role is taken by plasma membrane channels.

Moreover, the outcomes of apoptosis and necrosis can be explained without this assumption, by deleting equation 33 (Table 1) which models level 2 pore opening via sustained [Ca^2+^]_i_ increase and by introducing the equation PMCh_t_ = PMCh_t+1_ prolonging the opening of plasma membrane channels over the simulation time. Figure 8 shows that already one supplementary longer time step of channel opening is enough to induce the sustained elevation of [Ca^2+^]_i_ necessary for trypsin activation and necrosis (equations 39 and 41). Then, for the two basic stimulation conditions, with inhibition as well as with addition of antioxidants the dynamics and fit to the data is not changed (see also Additional file 2). Besides channel opening, other mechanisms of prolongation of high [Ca^2+^]_i_ can be imagined and could be investigated. In [6, p. 13130], the activation of different pathways by high and low stimulation with CCK is proposed, leading to the generation of the Ca^2+^ releasing messenger IP_3_ or only to the recruiting of active IP_3_ receptors.

However, ([2], Figure six A/B) gives a strong argument for ATP depletion under the investigated conditions: For a part of the cells stimulated with 200 μM TLC-S, [Ca^2+^]_i_ first oscillates followed by a monotonous increase (see Figure 2A). If ATP is added, the oscillations are sustained. Therefore, ATP decrease is the key process for a change from an oscillatory behaviour to a sustained [Ca^2+^]_i_ increase leading to trypsin activation and necrosis. As argued above, the opening of plasma membrane channels obviously is only necessary for a first extension of an elevated [Ca^2+^]_i_ concentration.

## Conclusions

The constructed logical model integrates heterogeneous processes like fast and slow molecular interactions or biological processes on a higher level, in order to explain dynamical and static data. The model of Ca^2+^ and ROS effects on apoptosis and necrosis, developed through several cycles of literature analysis and model adaptation, is able to generate realistic simulations during TLC-S stimulation, with and without the addition and inhibition of antioxidants. The variables Ca^2+^, ROS, NAD(P)H, apoptosis and necrosis exhibit the qualitative behaviour of the respective experiments. The analysis of the model demonstrates ROS effects in favour of apoptosis and the induction of necrosis via a sustained Ca^2+^ increase. The model provides the basis for further formal and experimental analyses through model-based predictions. An interesting example would be a direct experimental comparison of pancreatic and liver cells with respect to the ROS burst after mitochondrial pore opening observed in liver cells. Measurements of apoptosis and necrosis could provide further evidence for anti-inflammatory ROS effects.

Based on our previous work [32,18] in FCA, we presented here a novel application of the attribute exploration algorithm and of concept hierarchies to reveal dynamical features from data and simulations. The computed minimal and complete rule sets point at a gradual difference of the simulations and data regarding the fraction of apoptotic cells, if antioxidants are inhibited. In addition, the analysis reveals fine-grained differences in the succession of events. When focusing on interesting attributes, concept hierarchies offer a structured overview of dynamical features. For instance, this method highlighted the protective role of the highest ROS level (in the context of the analyzed bile stimulation) against necrosis. We here used attribute exploration as an open knowledge discovery method to unravel rules hidden in data and simulations. A complementary application of FCA, which we have not considered here, is to answer specific biological questions by querying the stem base.

The model explains how global [Ca^2+^]_i_ oscillations induced by medium bile stimulation promote ROS production, Cyt c release, activation of the caspase cascade and apoptosis. Increasing stimulation obviously does not influence these processes much. Instead, a large fraction of the cells undergoes necrosis via a positive Ca^2+^ feedback probably caused by enhanced mitochondrial pore opening, breakdown of the membrane potential and of ATP production, which hinders clearance of Ca^2+^.

We implicitly considered the exchange of Ca^2+^ between the cytosol, the ER, ZG and the extracellular space by a phenomenological rule generating oscillations (Table 1 (2)). Furthermore, we checked that intra-mitochondrial Ca^2+^ follows the cytosolic Ca^2+^ concentration in the appropriate time scale ([2], Figure three A). Model refinements are, however, possible by extension to a compartmental model, including cytosol and mitochondria as explicit variables, potentially also the ER, the ZG and the cell environment. This would allow a study of transport processes of Ca^2+^ as well as of NAD(P)H and antioxidants.

Also within our modelling framework, interesting biological questions still remain open. Theoretically, the model structure does not require ATP depletion. Instead, a sufficient long opening of plasma membrane channels could produce the same sustained Ca^2+^ increase. Even though the ATP depletion hypothesis is widely accepted and strong experimental evidence is given, the conflicting evidence from older experiments by [6] is still there.

Another testable hypothesis implied by the model is a self-enhancing cycle of Cyt c release and ROS production by interruption of the electron transport. This positive feedback should be activated by a primary ROS increase via Ca^2+^ during one (or several) cell-wide Ca^2+^ oscillations, which have a frequency of approximately one per minute. There is evidence for single interactions of this hypothesis [10,28], which again suggests further experimentation.

In conclusion, this study contributes to a more complete picture of ROS regulation and effects, beyond its predominant negative role as a cell damaging agent within the classical free radical theory of ageing. Our analysis, combining logical modelling and FCA, describes how ROS shift the balance from necrosis to apoptosis at the onset of acute pancreatitis and thus counteract the spreading of inflammation. 

R scripts for simulations and FCA analyses see www.sbi.uni-rostock.de/resources/software/multi-valued-logic-and-fca/.

In addition, the model was reimplemented with GINsim [33], exported to SBML-qual [34] and deposited in BioModels Database [35] with the identifier MODEL1407230001. Since SBML-qual does not support a priority update rule for the functions for a higher level of a variable, the logical functions were extended in order to exclude concurrency. Furthermore, a temporization like the update of three variable classes at different time scales (Methods) is not yet possible in SBML-qual. Instead, two priority classes were defined, which resulted in similar, but not identical dynamical features of the exported model. For more details see the BioModels annotation or the above web page describing the original model.

## Additional files

Additional file 1:
**BoothDataDiscrete.csv: **Discretized data from [2]. Additional file 2:
**Simulations.zip: **Tabular representation of simulations (i.) starting from normal physiological conditions without, with low and high bile stimulation (with supplementary plots); (ii.) with inhibition or addition of antioxidants; (iii.) prediction of the behaviour of liver cells; (iv.) without the assumption of ATP depletion. Additional file 3: Table S1.
**docx:** Discretized Ca^2+^, NADH and ROS measurements during 200 μM bile stimulation [2], transformed to a transition context. Additional file 4:
**AttributeExploration.zip: **The formal contexts *K*_sim_, *K*_obs_ and *K*_com_ for simulations, data and both, together with their dynamical rules generated by attribute exploration. Additional file 5:
**ROSInflammation_Network.xls: **References for the network interactions and the logical functions.

## Abbreviations

ATPase: (Ca^2+^-)ATP synthase
CCK: Cholecystokinin
[Ca^2+^]_i_: Intracellular calcium concentration
Cyt c: Cytochrome c
DMN: 2,4-dimethoxy-2-methylnaphtalene
ER: Endoplasmatic reticulum
ETC: Electron transport chain
FCA: Formal Concept Analysis
FRTA: Free radical theory of ageing
IP_3_: Inositol trisphosphate
NAC: N-acetyl-l-cysteine
NAD(P)H: Reduced nicotinamide adenine dinucleotide (phosphate)
MPTP: Mitochondrial permeability transition pores
NQO1: NADPH:quinone oxidoreductase 1
ODE: Ordinary differential equations
PI: Propidium iodide
ROS: Reactive oxygen species
TLC-S: Taurolithocholic acid sulfate
ZG: Zymogen granula
