# Supplementary figures and images for: Anti-inflammatory effects of reactive oxygen species – a multi-valued logical model validated by formal concept analysis
Source: BMC Syst Biol. 2014 Sep 24;8:101. doi: 10.1186/s12918-014-0101-7 (PMC4229622; doi:10.1186/s12918-014-0101-7)

Level

3

2

1

0

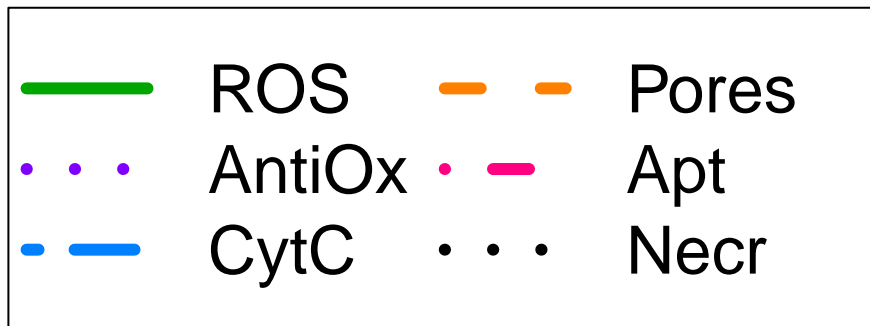

0

10

20

30

40

State

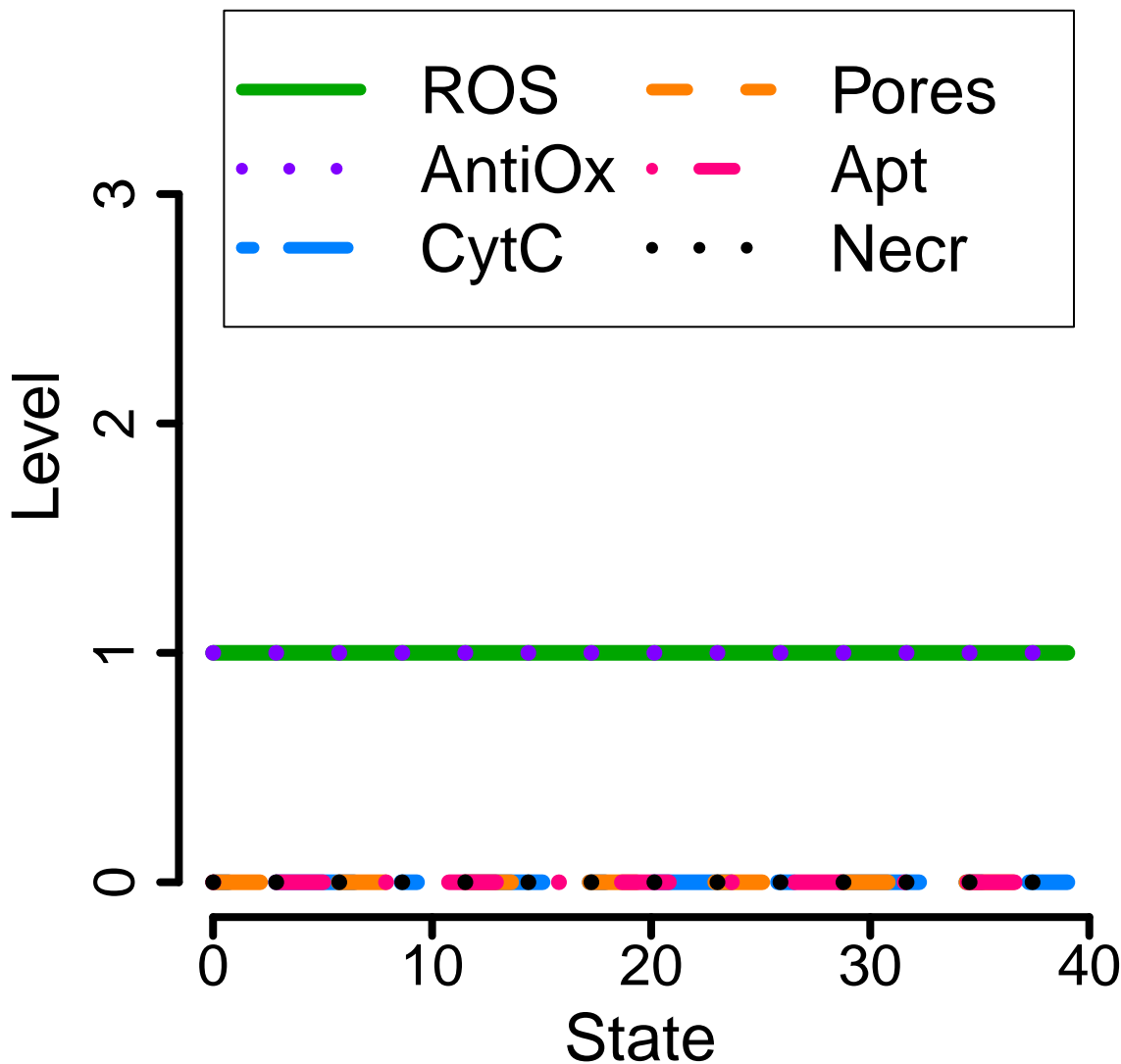

Supplement: Additional file 2: — Simulations.zip: Tabular representation of simulations (i.) starting from normal physiological conditions without, with low and high bile stimulation (with supplementary plots); (ii.) with inhibition or addition of antioxidants; (iii.) prediction of the behaviour of liver cells; (iv.) without the assumption of ATP depletion. [file 12918_2014_101_MOESM2_ESM.zip › Simulations/ROSInflammation_sim1b.pdf]

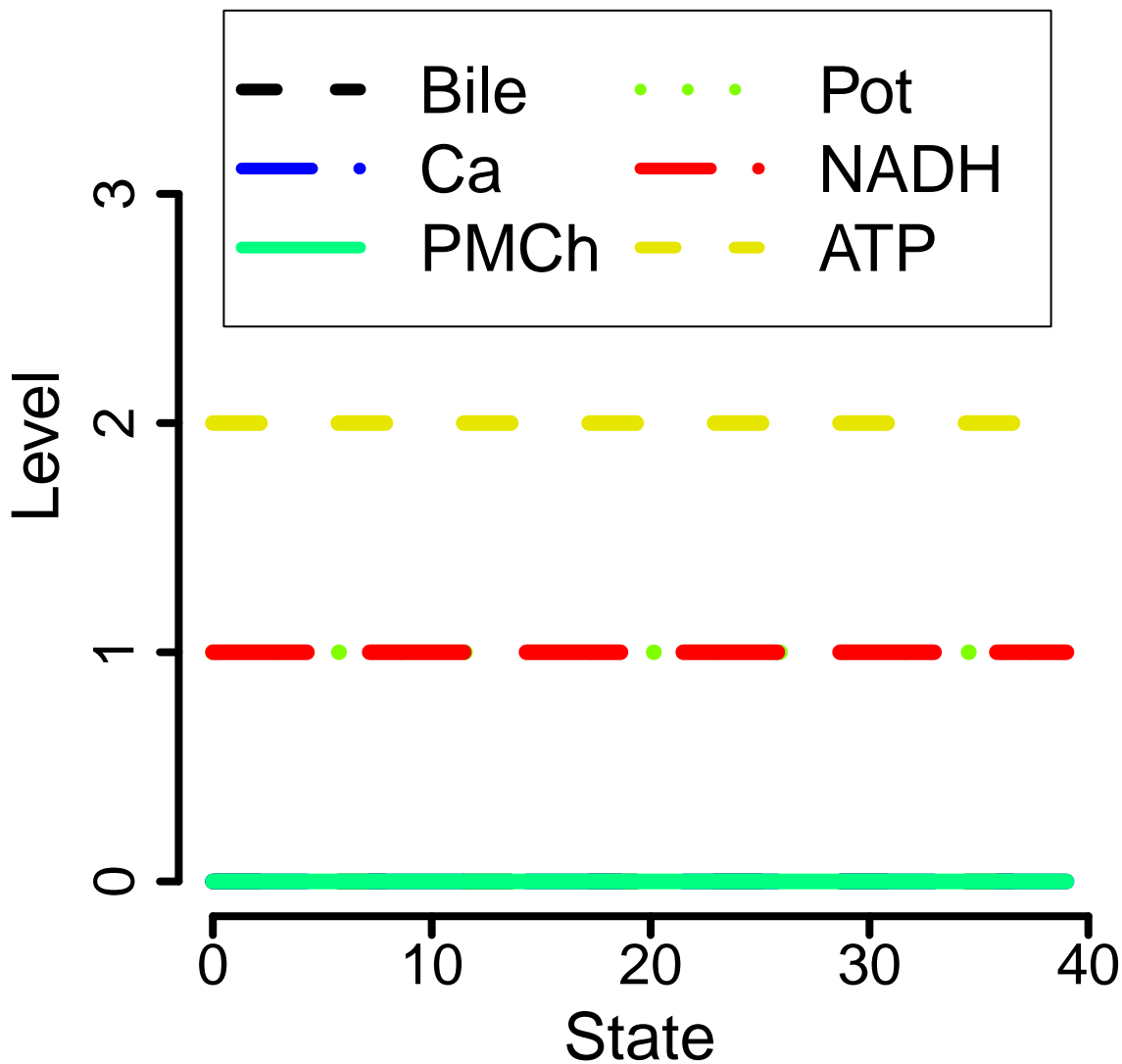

Supplement: Additional file 2: — Simulations.zip: Tabular representation of simulations (i.) starting from normal physiological conditions without, with low and high bile stimulation (with supplementary plots); (ii.) with inhibition or addition of antioxidants; (iii.) prediction of the behaviour of liver cells; (iv.) without the assumption of ATP depletion. [file 12918_2014_101_MOESM2_ESM.zip › Simulations/ROSInflammation_sim1a.pdf]

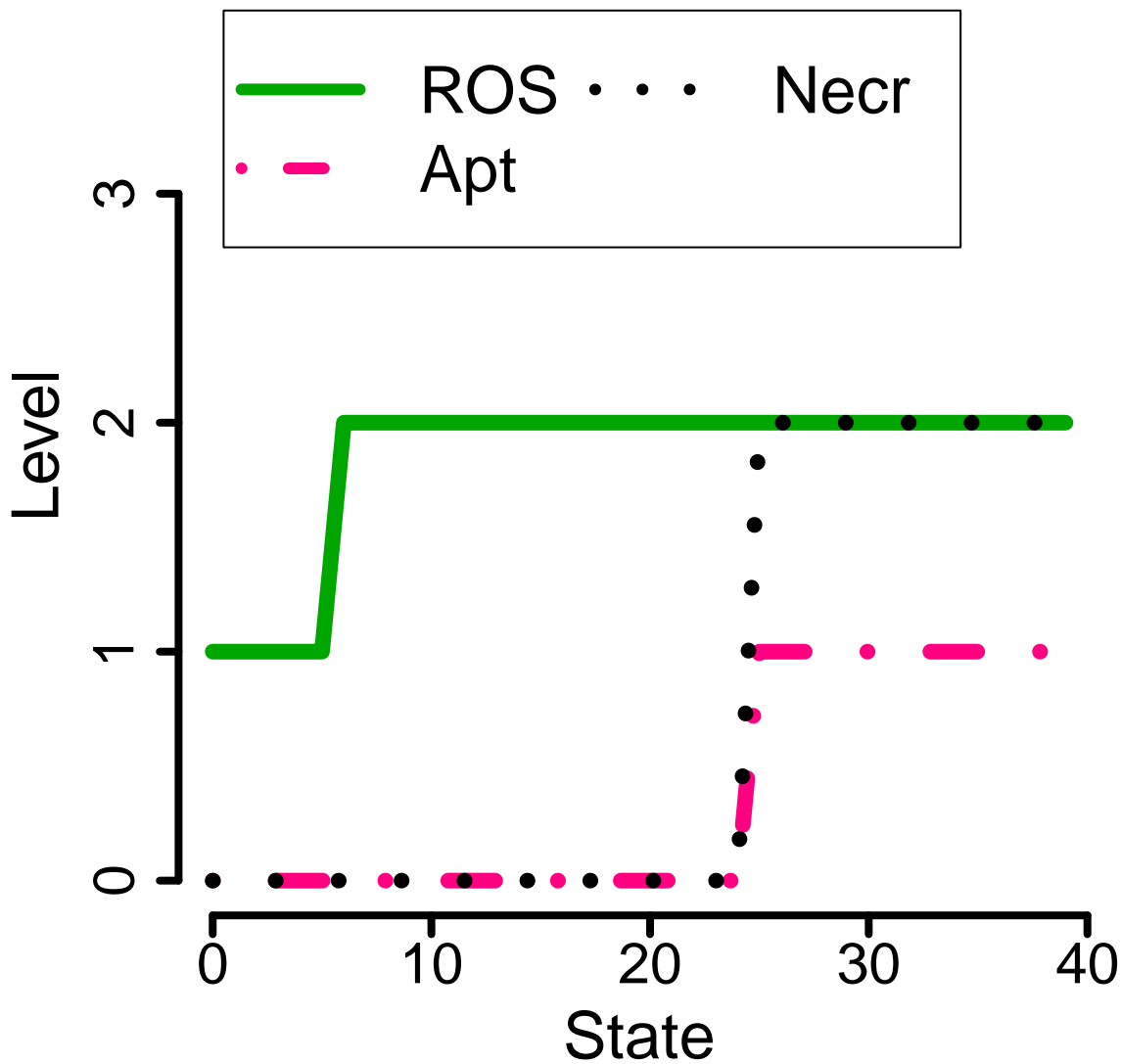

Supplement: Additional file 2: — Simulations.zip: Tabular representation of simulations (i.) starting from normal physiological conditions without, with low and high bile stimulation (with supplementary plots); (ii.) with inhibition or addition of antioxidants; (iii.) prediction of the behaviour of liver cells; (iv.) without the assumption of ATP depletion. [file 12918_2014_101_MOESM2_ESM.zip › Simulations/ROSInflammation_sim3_ROSEffects.pdf]

Level

3

2

1

0

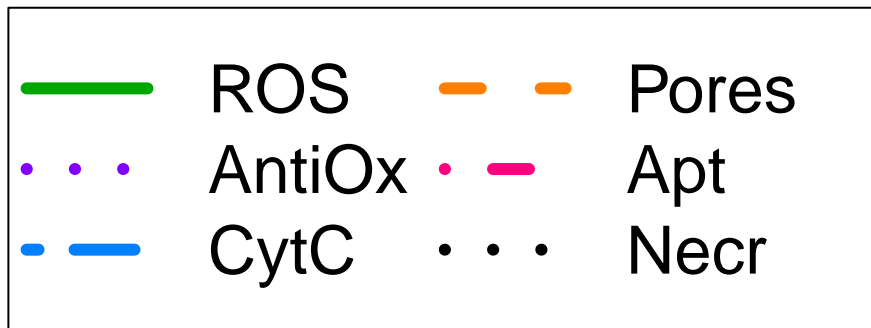

0

ROS

AntiOx

CytC

—

· · ·

- - -

Pores

· · ·

- - -

Apt

Necr

State

10

20

30

40

Supplement: Additional file 2: — Simulations.zip: Tabular representation of simulations (i.) starting from normal physiological conditions without, with low and high bile stimulation (with supplementary plots); (ii.) with inhibition or addition of antioxidants; (iii.) prediction of the behaviour of liver cells; (iv.) without the assumption of ATP depletion. [file 12918_2014_101_MOESM2_ESM.zip › Simulations/ROSInflammation_sim3b.pdf]

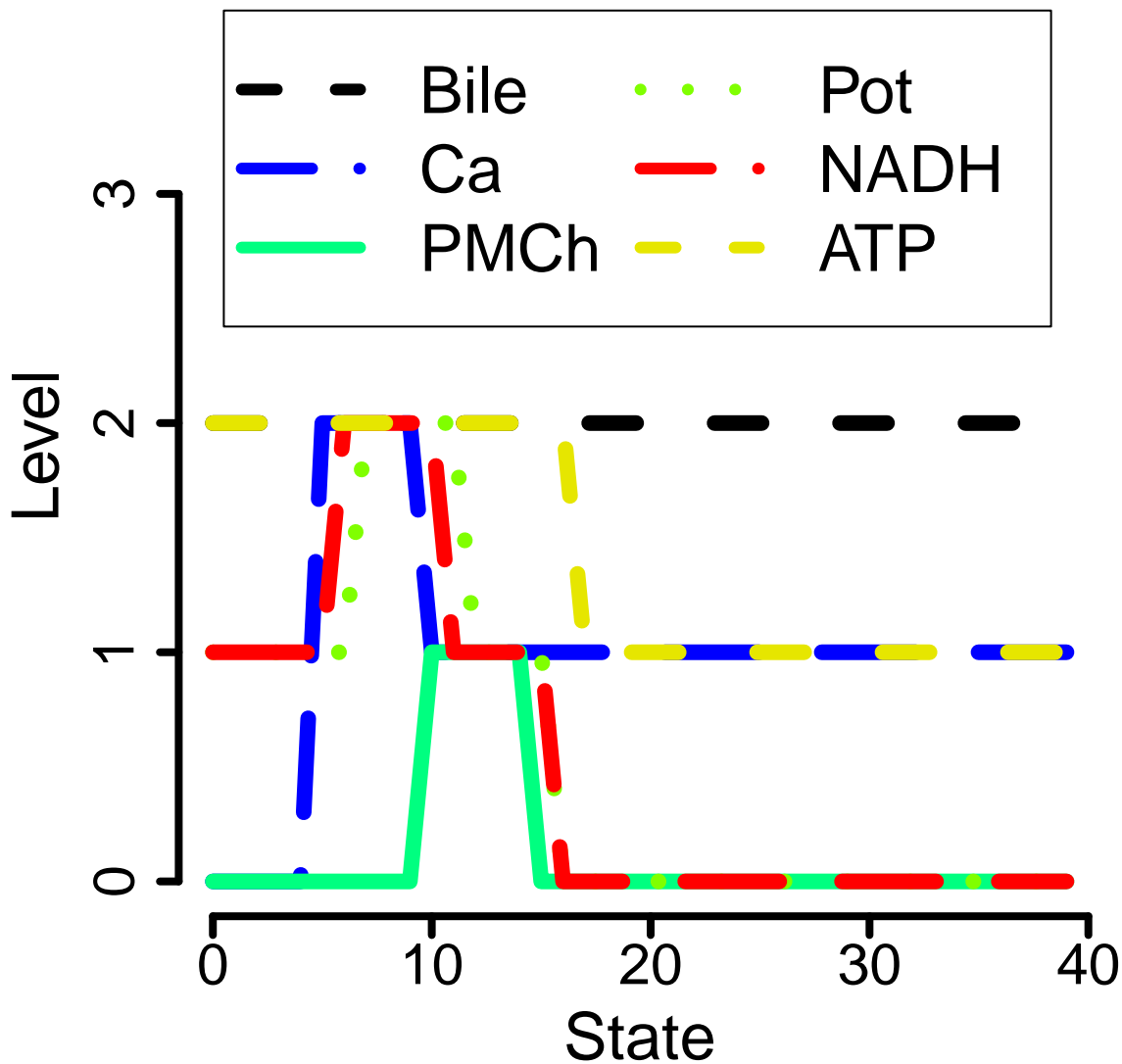

Supplement: Additional file 2: — Simulations.zip: Tabular representation of simulations (i.) starting from normal physiological conditions without, with low and high bile stimulation (with supplementary plots); (ii.) with inhibition or addition of antioxidants; (iii.) prediction of the behaviour of liver cells; (iv.) without the assumption of ATP depletion. [file 12918_2014_101_MOESM2_ESM.zip › Simulations/ROSInflammation_sim3a.pdf]

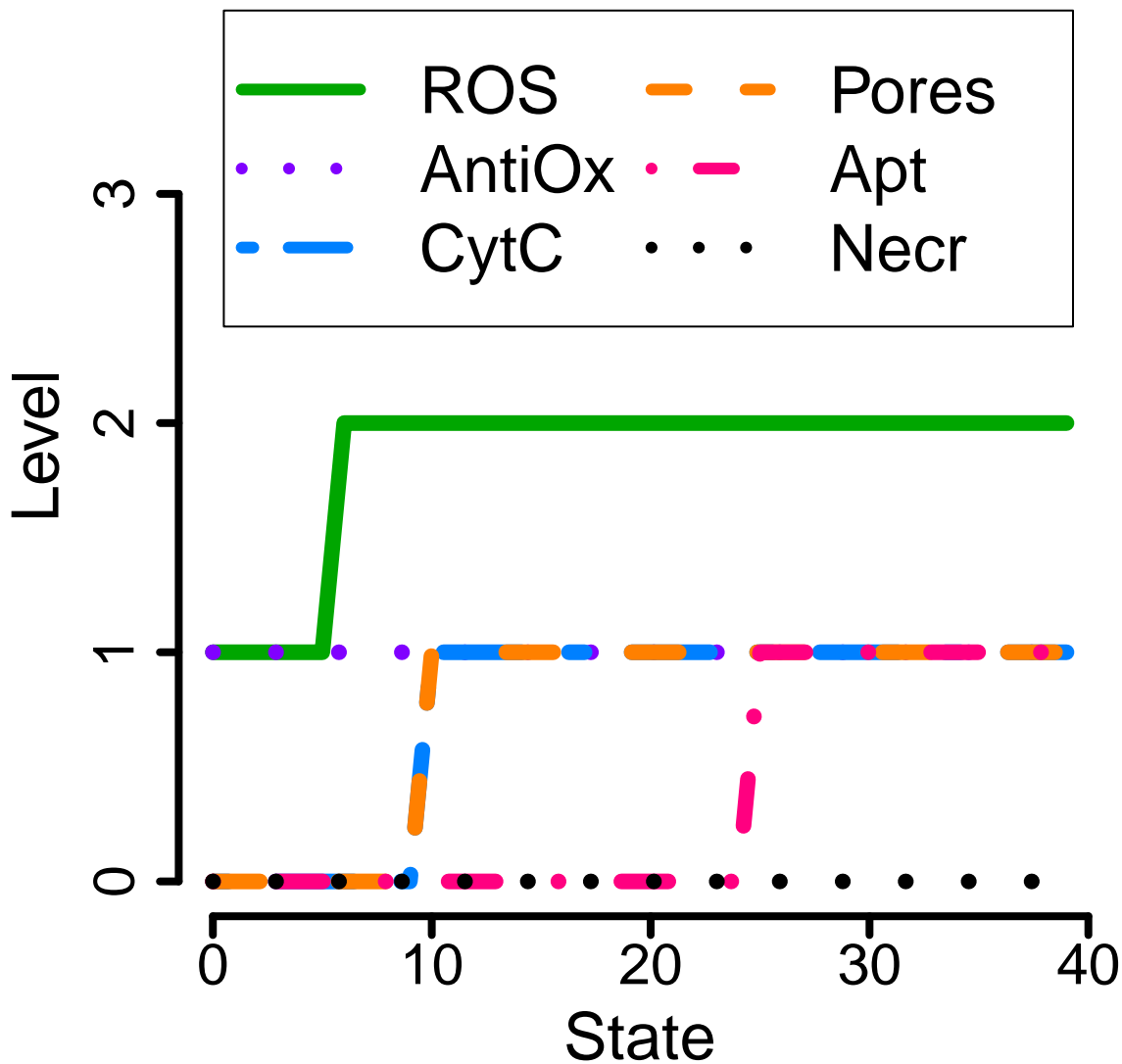

Supplement: Additional file 2: — Simulations.zip: Tabular representation of simulations (i.) starting from normal physiological conditions without, with low and high bile stimulation (with supplementary plots); (ii.) with inhibition or addition of antioxidants; (iii.) prediction of the behaviour of liver cells; (iv.) without the assumption of ATP depletion. [file 12918_2014_101_MOESM2_ESM.zip › Simulations/ROSInflammation_sim2b.pdf]

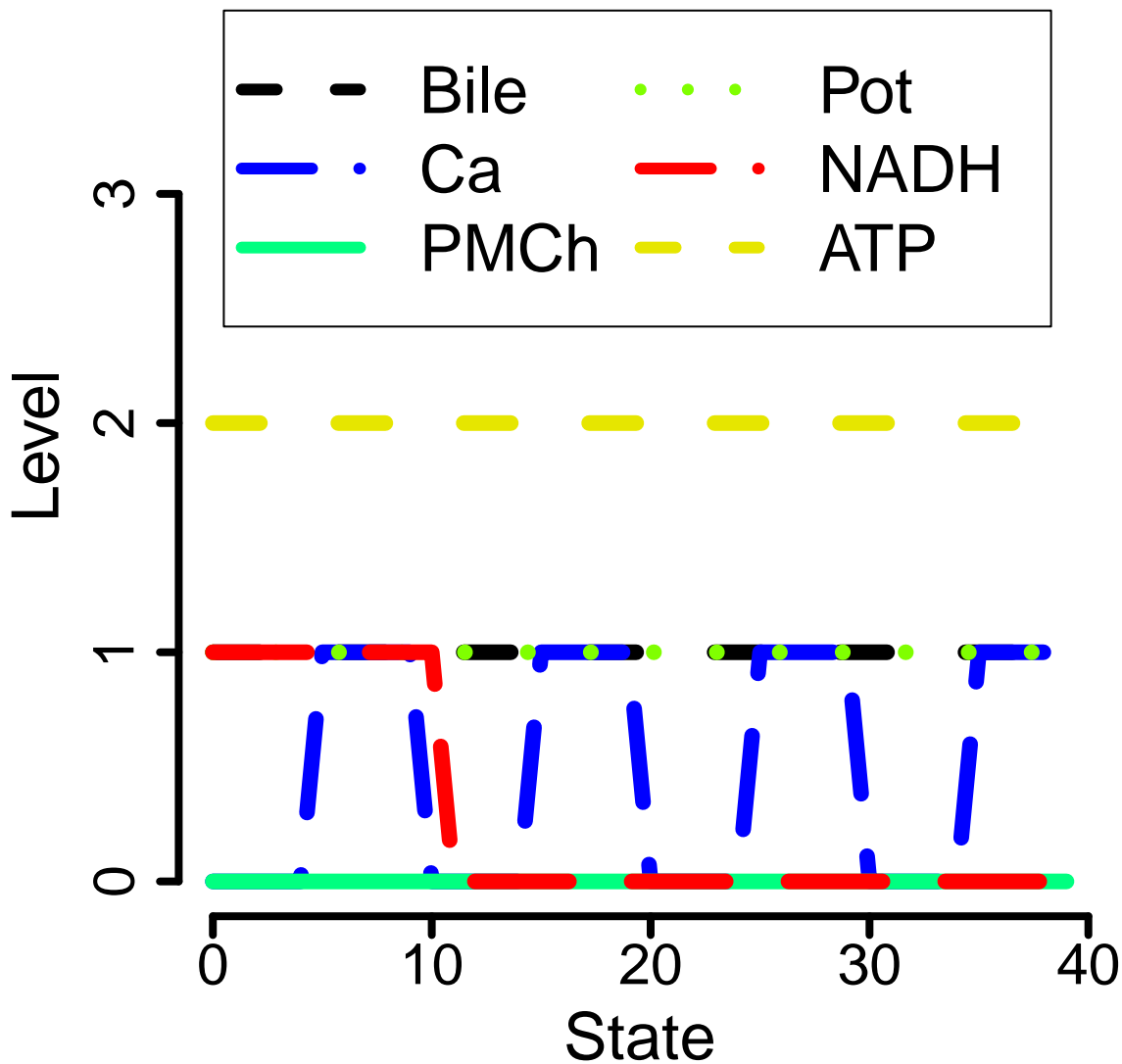

Supplement: Additional file 2: — Simulations.zip: Tabular representation of simulations (i.) starting from normal physiological conditions without, with low and high bile stimulation (with supplementary plots); (ii.) with inhibition or addition of antioxidants; (iii.) prediction of the behaviour of liver cells; (iv.) without the assumption of ATP depletion. [file 12918_2014_101_MOESM2_ESM.zip › Simulations/ROSInflammation_sim2a.pdf]
