# Supplementary material for: Anti-inflammatory effects of reactive oxygen species – a multi-valued logical model validated by formal concept analysis
Source: BMC Syst Biol. 2014 Sep 24;8:101. doi: 10.1186/s12918-014-0101-7 (PMC4229622; doi:10.1186/s12918-014-0101-7)
Supplement: Additional file 3: Table S1. — docx: Discretized Ca2+, NADH and ROS measurements during 200 μM bile stimulation [2], transformed to a transition context. [file 12918_2014_101_MOESM3_ESM.docx]

|  | **Ca.in** | **NADH.in** | **ROS.in** | **Ca.out** | **NADH.out** | **ROS.out** |
| --- | --- | --- | --- | --- | --- | --- |
| **(Bile1.0, Bile1.100)** | 0 | 1 | 1 | 1 | 1 | 2 |
| **(Bile1.100, Bile1.200)** | 1 | 1 | 2 | 0 | 1 | 2 |
| **(Bile1.200, Bile1.300)** | 0 | 1 | 2 | 1 | 1 | 2 |
| **(Bile1.300, Bile1.400)** | 1 | 1 | 2 | 0 | 0 | 2 |
| **(Bile1.400, Bile1.600)** | 0 | 0 | 2 | 1 | 0 | 2 |
| **(Bile1.600, Bile1.1200) BileBile1.1200)** | 1 | 0 | 2 | 0 | 0 | 2 |

**Table S1: Discretized Ca^2+^, NADH and ROS measurements in** [2]**, transformed to a transition context.** The rows represent transitions between states occurring during 200 µM bile stimulation (Bile1.*), for the fraction of cells with pure Ca^2+^ oscillations. The transition between the states observed after 200 and 300 seconds (third row) is a counterexample to the rule ROS.in.2, NADH.out.1 → Ca.in.1, which is valid for the simulations.
